# Identification of RNA Binding Proteins Associated with Dengue Virus RNA in Infected Cells Reveals Temporally Distinct Host Factor Requirements

**DOI:** 10.1371/journal.pntd.0004921

**Published:** 2016-08-24

**Authors:** Olga V. Viktorovskaya, Todd M. Greco, Ileana M. Cristea, Sunnie R. Thompson

**Affiliations:** 1 Department of Microbiology, University of Alabama at Birmingham, Birmingham, Alabama, United States of America; 2 Department of Molecular Biology, Princeton University, Princeton, New Jersey, United States of America; University of North Carolina at Chapel Hill, UNITED STATES

## Abstract

**Background:**

There are currently no vaccines or antivirals available for dengue virus infection, which can cause dengue hemorrhagic fever and death. A better understanding of the host pathogen interaction is required to develop effective therapies to treat DENV. In particular, very little is known about how cellular RNA binding proteins interact with viral RNAs. RNAs within cells are not naked; rather they are coated with proteins that affect localization, stability, translation and (for viruses) replication.

**Methodology/Principal Findings:**

Seventy-nine novel RNA binding proteins for dengue virus (DENV) were identified by cross-linking proteins to dengue viral RNA during a live infection in human cells. These cellular proteins were specific and distinct from those previously identified for poliovirus, suggesting a specialized role for these factors in DENV amplification. Knockdown of these proteins demonstrated their function as viral host factors, with evidence for some factors acting early, while others late in infection. Their requirement by DENV for efficient amplification is likely specific, since protein knockdown did not impair the cell fitness for viral amplification of an unrelated virus. The protein abundances of these host factors were not significantly altered during DENV infection, suggesting their interaction with DENV RNA was due to specific recruitment mechanisms. However, at the global proteome level, DENV altered the abundances of proteins in particular classes, including transporter proteins, which were down regulated, and proteins in the ubiquitin proteasome pathway, which were up regulated.

**Conclusions/Significance:**

The method for identification of host factors described here is robust and broadly applicable to all RNA viruses, providing an avenue to determine the conserved or distinct mechanisms through which diverse viruses manage the viral RNA within cells. This study significantly increases the number of cellular factors known to interact with DENV and reveals how DENV modulates and usurps cellular proteins for efficient amplification.

## Author Summary

DENV is the most important mosquito transmitted viral disease in humans causing almost 400 million infections each year. Early detection is possible; however, there are no antivirals or vaccines available for this potentially lethal virus. Host factors that are required for viral amplification provide an attractive target for antiviral therapeutics for rapidly evolving RNA viruses. However, only a handful of DENV host factors are known. This study reports identification of 79 novel dengue RNA binding proteins using a large-scale analysis of cellular proteins that interact with the DENV RNA during a live infection in human cells. Importantly, this analysis proved to have a remarkably low false discovery rate and the confirmed host factors appear to be specific to DENV without compromising the fitness of the cell for viral amplification of other unrelated viruses. Furthermore, these host factors were shown to be required at both early and late stages of the viral life cycle, consistent with the dynamic nature of dengue viral RNA.

## Introduction

Dengue is a mosquito-borne viral disease that infects 50–100 million people annually, resulting in dengue fever that is either asymptomatic or flu-like. However, tens-of-thousands of people develop the more severe, and sometimes fatal, dengue hemorrhagic fever/shock syndrome (DHF/DSS) [1]. DENV is found in most tropical and many subtropical areas with more than 125 countries being endemic for DENV [2]. There is no approved vaccine or antiviral therapeutic available for this life-threatening disease. Given the seriousness of infection, the expanding geographical range of the DENV, and the limitations in the existing measures of control and prevention, there is a pressing need to better understand the biology and pathogenesis of DENV.

DENV is a single-stranded positive-sense RNA virus that belongs to the *Flaviviridae* family. It has a 5’ cap, no poly(A) tail, highly structured 5’- and 3’- untranslated regions (UTR), and a single open reading frame (ORF) [reviewed in [3]]. Following virus entry, the viral RNA is released into the cytoplasm. Viral translation and replication occur in membranous assembly “factories” localized in the perinuclear region of endoplasmic reticulum (ER) [4]. The positive-stranded RNA molecules are encapsidated; virions are further processed as they are transported through the secretory pathway to the cell surface and released extracellularly [reviewed in [3]]. In addition to the viral proteins, cellular proteins, termed host factors, participate in most, if not all, steps of the DENV life cycle, including entry, translation, replication, virion assembly, and release [5]. Since viruses require host factors for efficient amplification, targeting host factors can provide an effective antiviral target for which the virus has no genetic control over. Therefore, it may be more difficult for viruses to evolve escape mutants that can replicate efficiently in the absence of the host factor [5, 6].

Several cellular proteins are known to impact DENV infection. For example, the polypyrimidine-tract-binding protein (PTBP1) is relocalized from the nucleus to the cytoplasm following DENV infection where it enhances DENV amplification by binding to the DENV 3’UTR and to NS4A, a viral protein required for the formation of the viral replication complex [7–10]. PTBP1 may also stimulate DENV translation [8], although this is still controversial [7, 9]. While most of the known DENV RNA binding proteins enhance viral amplification, several reduce DENV titers [10–12]. One such factor, YBX1, inhibits viral translation [12]. Although previous studies have laid a foundation for establishing critical interactions between viral RNA and cellular proteins [[13] and reviewed in [14]], the host factors identified thus far likely represent only a fraction of the total network of DENV host factors.

Previously, we have described a high-throughput mass spectrometry method termed TUX-MS (thiouracil cross-linking mass spectrometry) to identify host factors that interact with viral RNA during a live infection in cell culture [15]. Importantly, TUX-MS allows for identification of proteins that are bound directly to the viral RNA in living cells. Briefly, during a viral infection in cell culture, thiouridine is biosynthetically incorporated into the viral RNA to serve as a zero-distance cross-linker upon exposure to ultraviolet (UV) light. Thus, proteins that are bound directly to the viral RNA during a live infection are cross-linked to the RNA prior to disruption of cellular compartmentalization. This is particularly valuable for the identification of DENV host proteins, since DENV amplification is tightly associated with cellular membrane structures [4]. Following cross-linking, the viral RNA together with cross-linked proteins are isolated under denaturing conditions and identified by mass spectrometry-based proteomics. Using TUX-MS, we reported previously the successful identification and validation of host factors for poliovirus, pointing to a low false discovery rate of < 12% [15].

Here, we expanded the TUX-MS methodology for use with other types of RNA viruses, and investigated RNA-protein interactions during DENV infection. We modified the method to use virus-specific DNA oligos to capture the viral RNA and cross-linked proteins. Furthermore, we used metabolic labelling with stable isotopes to accurately quantify relative protein levels. This quantitative thiouridine cross-linking mass spectrometry (qTUX-MS) analysis identified 79 novel host proteins, which were not previously shown to be involved in DENV infection. We placed these findings in the context of whole proteome changes upon DENV infection, and further validated and functionally analysed a subset of the novel DENV host factor candidates. Overall, validation of the qTUX-MS identified factors using secondary assays indicates a low rate of false positives (17%), suggesting that the majority of the other identified qTUX-MS factors may also play significant roles in DENV viral amplification.

## Methods

### Cell culture and viruses

HeLa^UPRT^ cells expressing uracil phosphoribosyltransferase (UPRT) were generated previously by transduction of HeLa cells (ATCC, CCL-2) with UPRT-gene containing lentivirus [15]. Huh7.5^UPRT^ cells were generated by transduction of Huh7.5 cells (a kind gift from Charles M. Rice, Rockefeller University) with the same lentiviral construct as in [15]. HeLa^UPRT^ and Huh7.5^UPRT^ cells were cultured at 37°C and 5% CO_2_ in complete Dulbecco’s modified minimum essential medium (DMEM) supplemented with 10% FBS (Fetal bovine serum; Atlanta Biologicals) and penicillin-streptomycin and grown with 1 mg/ml G418 (Sigma) to select for the UPRT gene; Huh7.5^UPRT^ cells were additionally supplemented with non-essential amino acids (Cellgro). For SILAC labelling Huh7.5^UPRT^ cells were passed 1:10 at least twice in SILAC DMEM (Thermo Scientific) with 10% dialyzed heat-inactivated FBS (Thermo), L-proline (200 mg/L) and penicillin-streptomycin, and either 50 mg/L ‘heavy’ (13C6 L-lysine and 13C6-15N4 L-arginine; Cambridge Isotope Laboratories, Inc.) or 40 mg/L ‘light’ L-lysine and L-arginine amino acids [16].

Dengue virus serotype 2 (DENV2), strain 16681 (Genebank Accession number U87411) was kindly provided by Dr. Robert Striker (University of Wisconsin-Madison). DENV2 was propagated in the mosquito C6/36 cells at 28°C and 5% CO_2_ in advanced DMEM supplemented with 10% FBS, penicillin-streptomycin (Cellgro), L-glutamate and 10% tryptose phosphate broth (20 g/L tryptose; 2 g/L glucose; 5 g/L sodium chloride and 2.5 g/L disodium hydrogen phosphate). Titers for DENV2 were determined using plaque assays in BHK cells. For infections, cells were incubated with virus containing media for 2 hours, washed twice with the DMEM media after removal of the virus and incubated in the serum-free DMEM for the indicated time. Infections and titer determination of adenovirus 5 (Ad5) were performed exactly as in [15].

### Labelling of the magnetic beads

The DENV antisense biotin-labelled DNA fragments were generated using PCR and primers listed in (S1 Table) from the DENV2 complementary DNA (cDNA) and correspond to positions 4350–4914 and 4740–4914 of DENV genome. PCR was followed by removal of the unlabelled DNA strand according to the NanoLink Streptavidin Magnetic beads (Solulink) manual. The mixture of two biotinylated single-stranded DNA fragments of 174 base pairs (bp) and 564 bp long were bound to NanoLink Streptavidin Magnetic beads magnetic beads according to the manufacturer’s protocol.

### qTUX-MS

1–3 X 10^7^ human hepatoma Huh7.5^UPRT^ cells labelled with either ‘light’ or ‘heavy’ SILAC media were infected with DENV2 (MOI = 10) or mock-treated, respectively. Then, virus was replaced with SILAC media with 1 mM 4-thiouracil and 10% FBS. At 28 hours post-infection (hpi) the cells were washed with PBS and irradiated at 365 nm UV light for 20 min, collected, cell pellets were frozen on dry ice and stored at -80°C. Cell pellets were lysed in the lysis buffer (50 mM Tris-HCl pH 8, 4 mM magnesium chloride, 150 mM sodium chloride, 0.1% Tween-20, 5 mM dithiothreitol, Recombinant RNasin Ribonuclease Inhibitor [500 units/ml; Promega], 1× cOmplete protease inhibitor cocktail [Roche]), with a half volume of 465–600 μm glass beads (Sigma) by shaking at frequency of 30 Hz for 1 min using a Retsch MM200 mixer mill. An aliquot of ‘light’ and ‘heavy’ cell lysates were removed and the remaining lysates were incubated with the Streptavidin Magnetic beads labelled with DENV antisense DNA fragments for 15 to 30 min allowing for viral RNA to bind. The beads were washed twice with wash buffer I (50 mM Tris-HCl pH 8, 500 mM potassium chloride, 0.1% Tween-20), once with wash buffer II (50 mM Tris-HCl pH 8, 150 mM sodium chloride, 0.5% sodium deoxycholate) and once with 10 mM Tris pH 7.6. The samples were eluted at 65°C for 2 min in 20 μl of 10 mM Tris pH 8.0. The eluted ‘light’ and ‘heavy’ samples were mixed at a 1:1 ratio, RNA was degraded with 0.5 ng/μL bovine RNase A (Fisher Scientific) at 25°C for 24 hrs and subjected to quantitative mass spectrometry-based proteomic analysis.

### Quantitative mass spectrometry-based proteomic analysis of RNA-interacting proteins (qTUX-MS)

Protein eluates and their respective mixed light/heavy input lysates were subjected to in-solution enzymatic digestion using a filter-aided sample preparation approach [17], then analyzed by nLC-MS/MS, as described in S1 Methods and in [18].

### Quantitative mass spectrometry-based proteomic analysis of DENV-infected cells

Light (DENV2) and heavy-labelled (mock) cell pellets were lysed in 100 mM ABC containing 5% sodium deoxycholate at 95°C to ensure denaturation and virus inactivation. The protein concentrations were determined by the BCA assay and mixed in equal protein amounts (100 μg total). Proteins were subjected to in-solution digestion with trypsin, then fractionated and analyzed by nLC-MS/MS as described in the S1 Methods.

### Proteomic data analysis

qTUX-MS, its respective mixed input lysate, and the whole cell SILAC instrument raw data were separately processed using the MaxQuant software (ver. 1.5.3.8), configured with default settings, except for experiment-specific parameters, which are described in the S1 Methods. The mass spectrometry proteomics data have been deposited to the ProteomeXchange Consortium via the PRIDE [19] partner repository with the dataset identifier PXD003593.

### Bioinformatics analysis

Using the filtered list of protein identifications, unique gene symbols were used for downstream functional ontology analyses. The gene ontology annotations from UniProt were used to generate and assign the DENV2 RNA interacting candidates into broader functional categories. For the whole cell SILAC protein expression study, genes and their associated ratios were analyzed by PANTHER gene enrichment (PANTHER database ver 10.0, 2015-05-15) [20] using the PANTHER Pathway and Protein Class ontologies. Significant differential protein abundance was determined as a function of ontological classification versus the overall population (Bonferroni-corrected p-value < 0.05). For specific functional protein ontologies that were differentially regulated, a subset were selected for analysis by the Reactome Functional Interaction (FI) network Cytoscape plugin [21]. The Reactome FI plugin was used to visualize candidate host factors identified by qTUX-MS.

### siRNA transfections

2 X 10^6^ HeLa^UPRT^ cells were transfected with 350 pmol of Silencer Select Negative Control (Ambion) or the specified siRNAs (S1 Table) using the XtreamGENE siRNA Transfection Reagent system (Roche). 24 hrs later, 4 X 10^5^ cells/well were plated for infection. 48 hours post transfection cells were infected (MOI = 0.1) with DENV2 or Ad5 (n = 3). At either 30 (Ad5) or 40 (DENV2) hpi, virus titers were determined by plaque assays using 911 or BHK cells, respectively. Knockdown efficiency was measured 48 hrs post transfection by RT-qPCR. Experiments were performed in two biological replicates for each host factor. Statistical analysis was performed using student’s t-test.

### Cell viability and proliferation assay

24 hrs post siRNA transfection equal numbers of cells (either 2 X 10^3^ or 8 X 10^3^) were seeded in 96-well plates. Cell viability was measured 48 hours after siRNA-mediated knockdown of individual host factors using the Vybrant MTT [3-(4,5-dimethyl-2-thiazolyl)-2,5-diphenyl-2H-tetrazolium bromide] assay kit (Invitrogen) according to the manufacturer's protocol and reported relative to the negative-control siRNA (set to 100%; n = 3). Statistical analysis was performed using student’s t-test.

### Real-time qPCR analysis

cDNA was generated from 1μg TRIzol (Ambion) purified total RNA using Moloney murine leukemia virus (MMLV) reverse transcriptase (Promega) as described by the manufacturer using random primers (Invitrogen). qPCR was performed using iQ SYBR green Supermix (Bio-Rad) with the primers listed in the S1 Table. The amplification efficiency for each primer set was 100±10% as determined using a standard curve.

## Results

### Development of a quantitative thiouridine cross-linking mass spectrometry (qTUX-MS) method for identification of proteins associated with the DENV RNA

**Fig 1:**
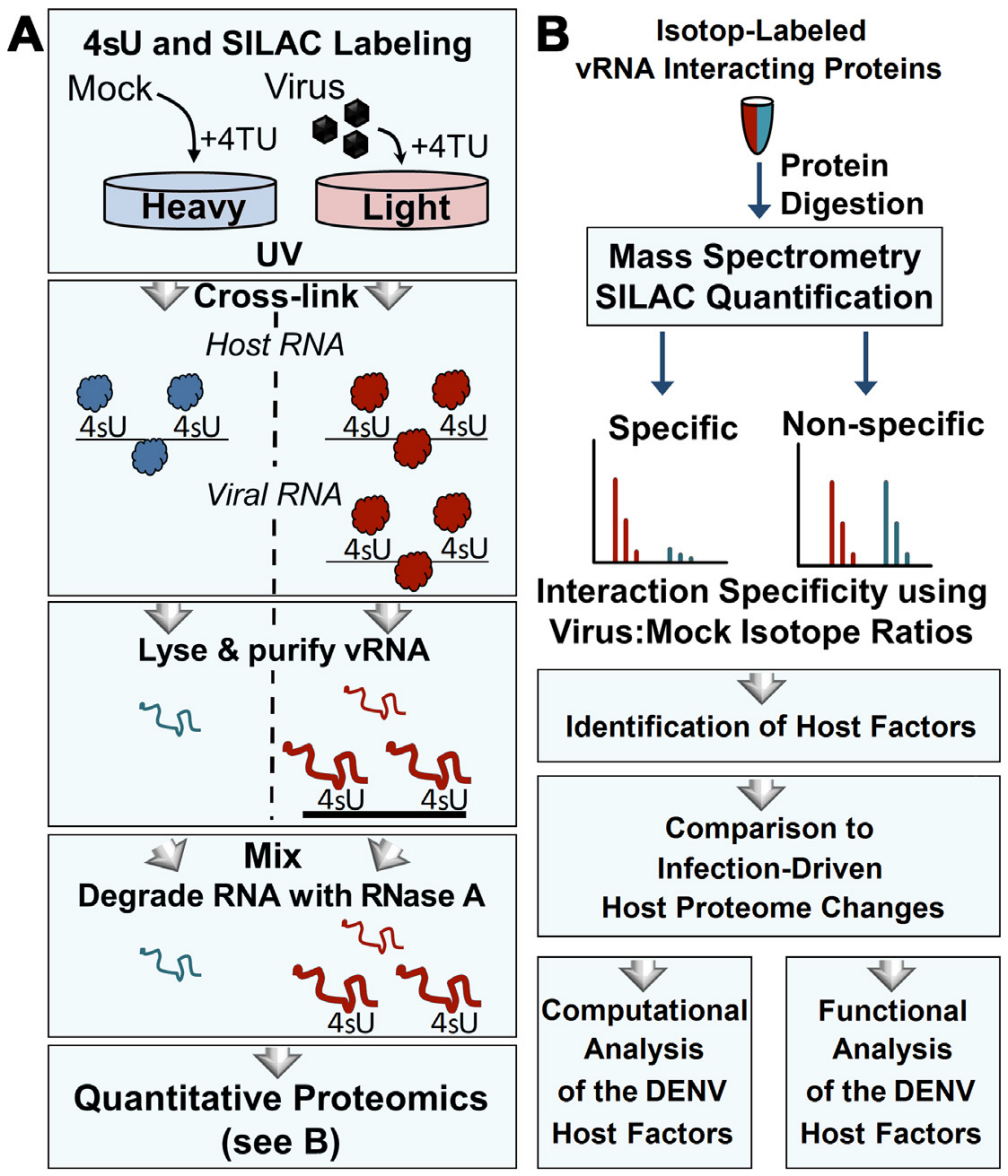
Isolation and identification of cellular proteins that associate with DENV RNA by qTUX-MS. (**A**) Diagram of the qTUX-MS method and SILAC labelling. Huh7.5^UPRT^ cells labelled with the ‘light’ (red) or ‘heavy’ (blue) amino acids are infected with DENV or treated with mock, respectively, in the presence of 4TU. 4sU is incorporated into cellular and DENV RNAs and proteins are UV cross-linked to the contacting thio-containing RNA (represented as either balls to indicate native conformation or curved lines to indicate denatured proteins) in living cells at 28 hpi prior to cell lysis under denaturing conditions. Viral ribonucleoprotein complexes were isolated using DNA molecules complementary to DENV RNA bound to magnetic beads, the RNA was degraded with RNase A and the proteins were identified by mass spectrometry. (**B**) Workflow for quantitative proteomic analysis of RNA-bound host factors isolated in (A). Isolated proteins were mixed between mock and virus-infected samples, digested into peptides, and analysed by mass spectrometry. Relative ‘light’ and ‘heavy’ peptide abundances were quantified to determine the specificity of interaction. Host factor candidates were identified and subjected to functional validation.

TUX-MS can be used to identify host factors by incorporating 4-thiouridine (4sU), a zero-distance cross-linker, into the viral RNA (vRNA) to enable cross-linking of proteins bound to vRNA during a live infection in cell culture [15]. Cross-linking is carried out under physiological conditions prior to cell lysis to ensure specificity and reduce false-positives from non-specific RNA protein interactions that occur upon loss of compartmentalization. vRNA is isolated under denaturing conditions and cross-linked proteins are identified using liquid chromatography-tandem mass spectrometry (LC-MS/MS). To improve quantification of the TUX-MS identified host proteins (qTUX-MS), a SILAC (stable isotope-labelled amino acids in cell culture) approach [16] was used to label the uninfected (mock) and infected cells with either ‘heavy’ or ‘light’ amino acids, respectively (Fig 1A). When 4-thiouracil (4TU) is present in the medium, Huh7.5^UPRT^ human hepatoma cell lines stably expressing UPRT (uracil phosphoribosyltransferase) convert 4TU to UMP. Then, the UMP is converted to thiouridine triphosphate (4sUTP) by cellular kinases [22]. Both cellular and viral RNA polymerases use 4sUTP as a substrate during RNA synthesis, which serves as a zero-distance cross-linker, covalently binding proteins to RNAs upon exposure to long wave UV-light. Importantly, protein-protein cross-linking is very inefficient at long UV wavelengths, ensuring that only proteins in direct contact with the reactive thiol group of the 4sU-containing RNA will be cross-linked [23]. We have shown previously that immunoisolation of candidate vRNA-binding proteins identified by TUX-MS (and confirmed by western) could be specifically co-isolated with viral RNA [15]. Together, this study established that TUX-MS can identify bona fide interactions between host proteins and viral RNA. The TUX-MS method was originally developed to capture polyadenylated RNA using oligo(dT) beads [15]. However, as DENV RNA is not polyadenylated, we modified the method to use sequence specific capture of the vRNA using magnetic beads. Following crosslinking in Huh7.5^UPRT^ cells infected with DENV at 28 hpi and affinity capture of the vRNA, the ribonucleoprotein complexes were eluted from the beads, ‘heavy’ and ‘light’ eluates were mixed, and RNase A was used to degrade the RNA. The proteins were digested in-solution with trypsin and subjected to quantitative MS-based proteomics (Fig 1B). The median ‘light’ to ‘heavy’ peptide and protein ratios were calculated, reflecting the specificity of vRNA-protein capture. We identified several classes of proteins, including DENV proteins, known DENV host factors, and putative RNA-interacting host proteins, but most of the qTUX-MS identified factors have not been previously identified through interactions with DENV (S2 Table).

### Viral proteins and known host factors were identified by qTUX-MS

Consistent with previous knowledge of flaviviral RNA [24–27], our qTUX-MS analysis identified several viral proteins—C, E, NS3, NS4A and NS5—as associated with vRNA. In addition, qTUX-MS identified six known DENV host factors: polypyrimidine tract-binding protein 1 (PTBP1), interleukin enhancer-binding factor 3 (ILF3), calreticulin (CALR), calnexin and heterogeneous nuclear ribonucleoproteins hnRNP H1 and hnRNP K, as well as a known DENV anti-viral protein—eukaryotic initiation factor 4A (eIF4A)—and 12 other proteins previously shown to associate with DENV RNA or proteins (S2 Table). Altogether, since several known host factors were identified using qTUX-MS, this suggests that the adapted method is effective at identifying host factors for DENV.

### Novel cellular factors were identified by qTUX-MS

For identification of novel host factors, cellular proteins with a DENV/Mock SILAC ratio of ≥ 1.5-fold (n ≥ 3 quantified peptides) were considered putative DENV vRNA interactions. This threshold was selected when considering the median variance in the SILAC ratio (for proteins with > 3 quantified peptides), which was approximately 25%. Therefore, we opted for a conservative cut-off at 50%, representing twice this median value or 1.5-fold. Common environmental and non-human cell culture contaminants were excluded, since they existed in only the ‘light’ SILAC state. In addition, our qTUX-MS samples also contained histones: H3, H4, H2A, H2B, H1.5 and macroH2A.1. In a previous study, histones were shown to play roles in dengue infection [28]. However, their functions were mediated through an interaction with a viral capsid protein and were shown to be independent of RNA. In addition, histones are primarily nuclear and highly abundant proteins commonly detected (> 50%) in control affinity purifications compiled across diverse protein-protein interaction studies [29]. For these reasons, histones likely represent non-specific associations rather than DENV RNA binding factors, and thus were excluded from further analysis. In total, 93 cellular proteins passed these selection criteria, 79 of which have not been previously shown to associate with DENV (S2 Table, S1 Dataset). Several of the known DENV host factors were enriched but did not meet the stringent inclusion criteria (S2 Table), suggesting that there may be additional host factors below our enrichment threshold (see S1 Dataset). Importantly, the subset of 79 host factors represents a significant potential expansion in the number of known DENV host factors, providing a valuable resource to test for pro-viral or antiviral activities during DENV infection.

### The majority of candidate host factors are not altered at the protein level upon DENV infection

**Fig 2:**
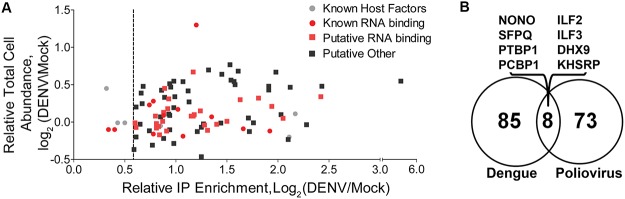
Comparison of DENV/MOCK qTUX-MS protein enrichment and whole cell expression. (**A**) Scatter plot comparison of the relative log_2_ total cell protein abundance versus the relative log_2_ qTUX-MS enrichment for 93 proteins quantified by qTUX-MS. Grey and red circles, known pro- & anti-viral factors and known vRNA-binding proteins, respectively; red and black squares, putative factors with RNA binding annotation and putative host factors with other annotation, respectively. (B) Venn diagram representing the overlap between the cellular factors identified to bind DENV RNA (this study) and the proteins previously described to bind poliovirus RNA [15]. The proteins found in both studies are listed above the diagram.

It is well recognized that viral infections can induce significant changes in cellular proteomes [30–32] and an increase in protein levels during DENV infection may contribute to the increased protein capture measured by qTUX-MS. To address this, we used SILAC-MS to quantify proteome (i.e., total protein abundance) changes following DENV infection. Comparison of qTUX-MS and proteome SILAC ratios showed that the protein abundances for the 93 qTUX-MS-identified vRNA-binding factors remained largely unchanged (Fig 2A, S1 Fig and S1 Dataset). On average, for these proteins, the DENV-induced changes in whole cell abundance were ±1.1-fold, suggesting that their identification by qTUX-MS was not due to an increase in their abundance in the cell following DENV infection. Noteworthy, a retrospective qualitative comparison of the qTUX-MS identified factors for DENV with those identified in the TUX-MS analysis on poliovirus revealed less than 10% were identified for both viruses [15] (Fig 2B). Since the identified proteins are largely DENV specific, qTUX-MS is not biased towards identifying a sub-set of cellular RNA binding proteins. Taken together, our results indicate that the enrichment ratios measured by qTUX-MS is predominantly due to their specific association with the DENV RNA.

### DENV infection induces changes in expression for proteins associated with molecular transporters and the ubiquitin proteasome system

**Fig 3:**
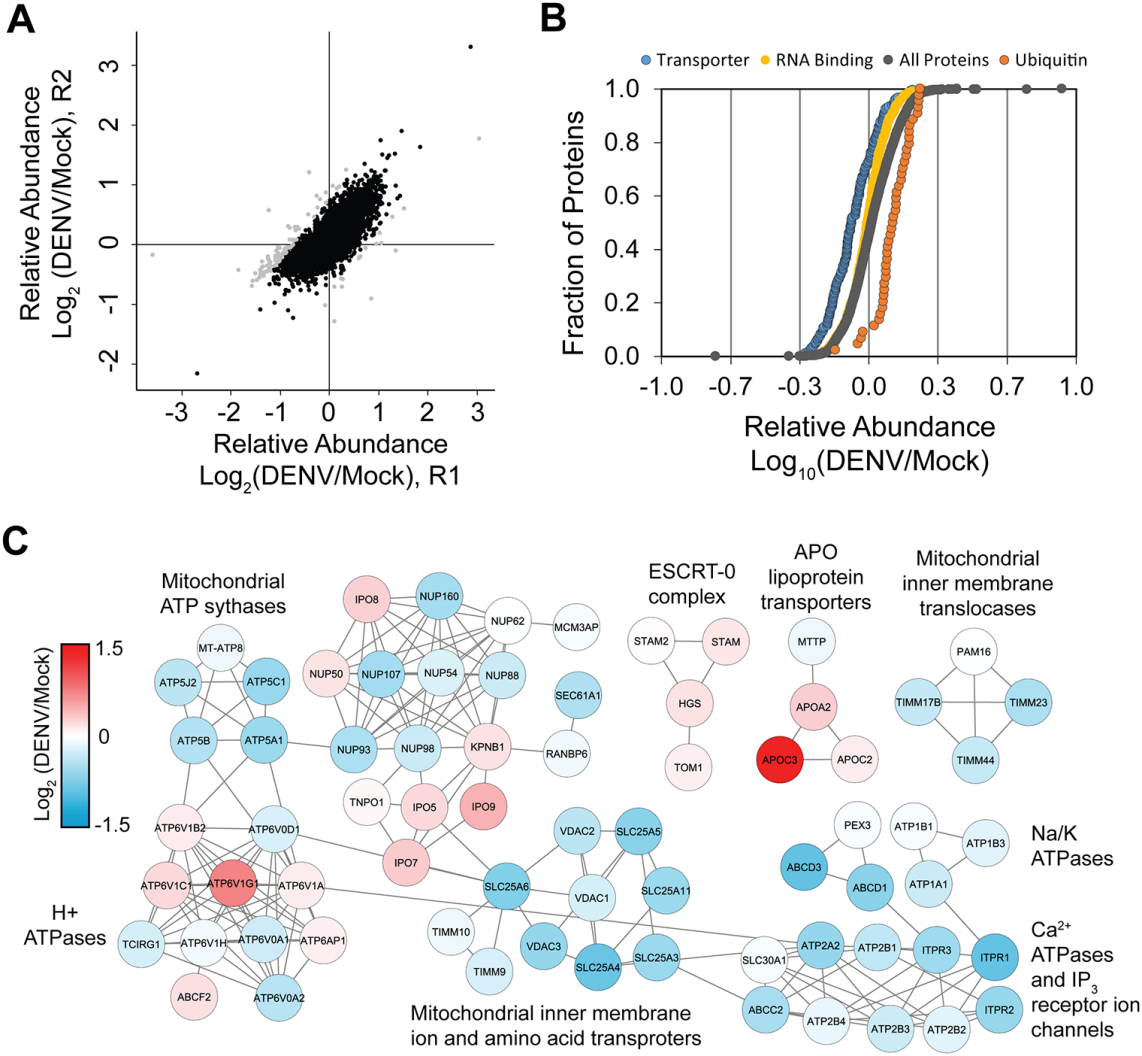
DENV virus infection induces differential expression of selected protein classes. (**A**) Comparison of 4,907 log_2_ DENV/Mock host protein abundance ratios between replicate experiments (R1 and R2). Black dots, proteins that were quantified with < 50% variance between replicates. (**B**) Comparison of log_10_ relative abundances of all host proteins (grey circles) to proteins annotated in the Transporter (blue circles, N = 209), RNA binding (yellow circles, N = 522), and ubiquitin proteasome pathway (orange circles, N = 44). Abundance ratios are plotted in increasing magnitude and expressed as a fraction of the total number of proteins. (**C**) Reactome functional interaction networks of proteins annotated in the transporter PANTHER protein class ontology. Four sub-networks (≥ 3 proteins per network) containing 71 proteins were formed. Nodes labeled with gene symbols and colored by relative log2 protein abundance. Clusters are labeled with representative transporter classes.

While proteins that bound DENV RNA did not show significant changes in abundance upon infection, we performed bioinformatics analysis on the complete proteome dataset of whole cell abundance to determine the global proteome effects of DENV infection under these conditions. In total, 4,907 host proteins were quantified by SILAC MS in biological duplicates (S1 Dataset). The abundance ratios between biological duplicates were reproducible, with only ~ 2% of the ratios varying by > 50% (Fig 3A). From these duplicates, an average abundance ratio was calculated and the respective proteins were analyzed by PANTHER gene enrichment analysis (S2 Fig) [20]. This analysis found systematic up regulation of proteins in the ubiquitin proteasome pathway (UPP), comprising 18 members of the 26S proteasome as well as various ubiquitin-conjugating enzymes (S3 Fig). Many of these enzymes are linked to ubiquitin-dependent proteasome degradation, consistent with the current knowledge that the UPP is important for production of infectious virions [31, 33, 34]. Yet, other enzymes, such UBE2N and UBE2V2, catalyze polyubiquitination at Lys-63, which does not lead to proteasome degradation but rather participates in transcriptional activation of target genes and may promote innate immune signaling [35, 36]. In contrast, proteins in the transporter protein class were on average down regulated (Fig 3B and S2 Fig). Assembly of the annotated proteins into Reactome protein networks identified several subnetworks with various transporter activities (S4 Fig). While the abundances of mitochondrial transporters and nucleoporins were the most consistently decreased, not all transporters were down regulated; for example, the lipoprotein (APO) transporters were increased in expression (Fig 3C). Interestingly, the RNA binding protein class was significantly down regulated (Fig 3B and S2 Fig), though the effect was not as pronounced as the transporter class (Fig 3B). The overall down-regulation of RNA binding proteins appears to be driven by changes in cytoplasmic and mitochondrial ribosomal subunits, and proteins involved in RNA degradation and processing (S5 Fig). Nevertheless, the relative protein abundance for the set of 93 (known and putative) DENV binding factors identified by qTUX-MS was largely unchanged, despite being enriched in RNA processing and translation factors (Fig 2A). Overall, the quantitative proteome analysis suggests that DENV selectively alters the abundance of proteins, and reveals several pathways that could be directly or indirectly modulated in the host response to DENV infection.

### Reactome analyses of qTUX-MS identified factors reveals a core network of RNA and DNA binding activities

**Fig 4:**
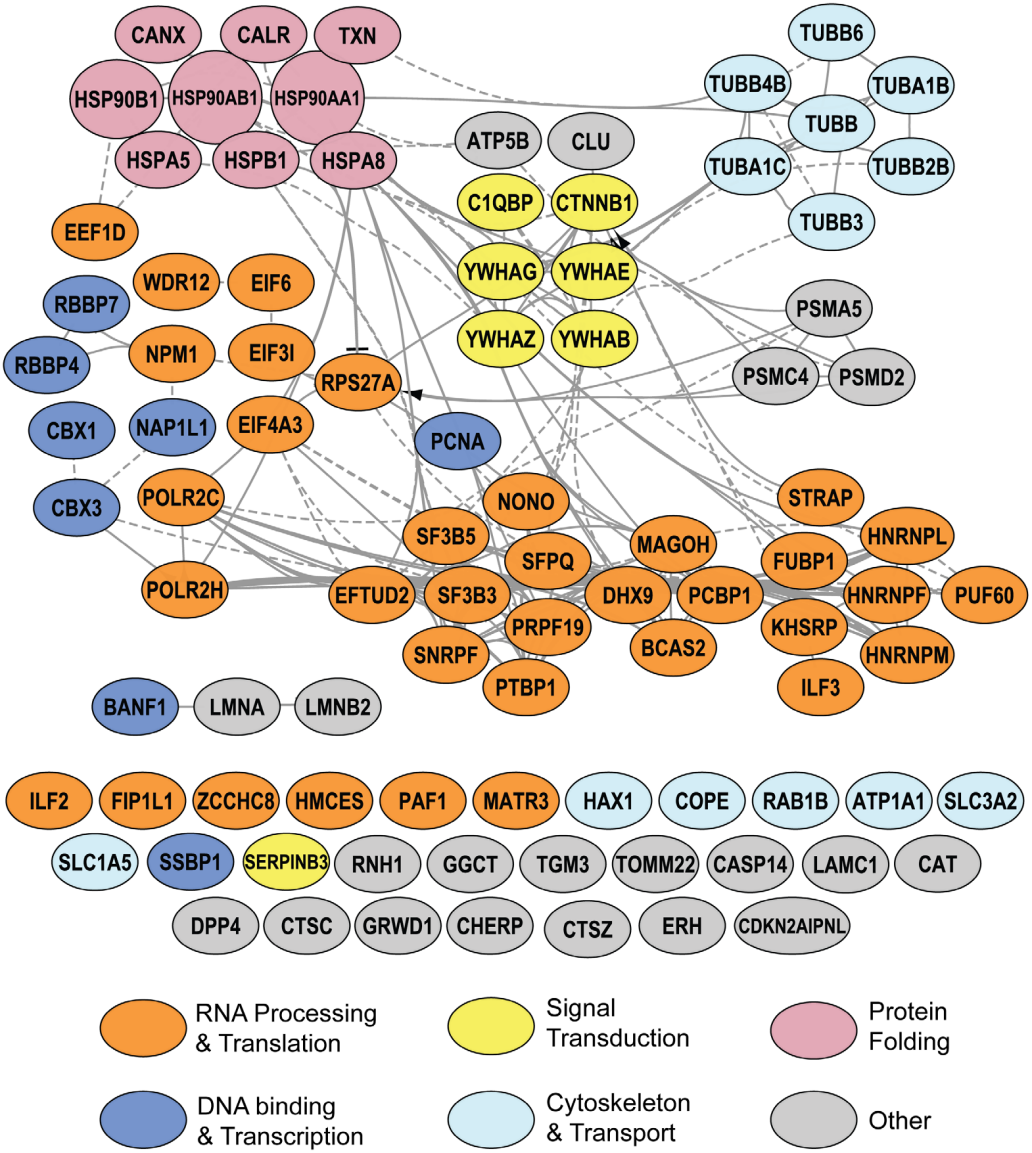
Functional network of known and putative host factors interacting with DENV RNA identified by qTUX-MS. Ninety-three host proteins identified by qTUX-MS were analyzed by the Reactome FI Cytoscape plugin. Network nodes are labeled with gene symbols and color-coded by broad functional classes

To gain insight into potential molecular mechanisms and biological processes of the 93 qTUX-MS identified factors, we performed a functional network-based analysis using the curated human pathway relationships from the Reactome database. This analysis revealed a high degree of connectivity, with 62 proteins forming a large interconnected network (Fig 4). The densest network connectivity included proteins involved in RNA Processing/Translation (orange nodes) and DNA binding/Transcription (blue nodes). Several additional proteins with RNA and/or translational activities were also identified, but lacked annotation in Reactome (orange single nodes). Overall, our bioinformatic evaluation further supports the ability of qTUX-MS to capture vRNA-bound host factors and points to their possible function in DENV amplification.

### RBMX, NONO, hnRNP M, hnRNP F and HMCES are required for efficient amplification of DENV

**Fig 5:**
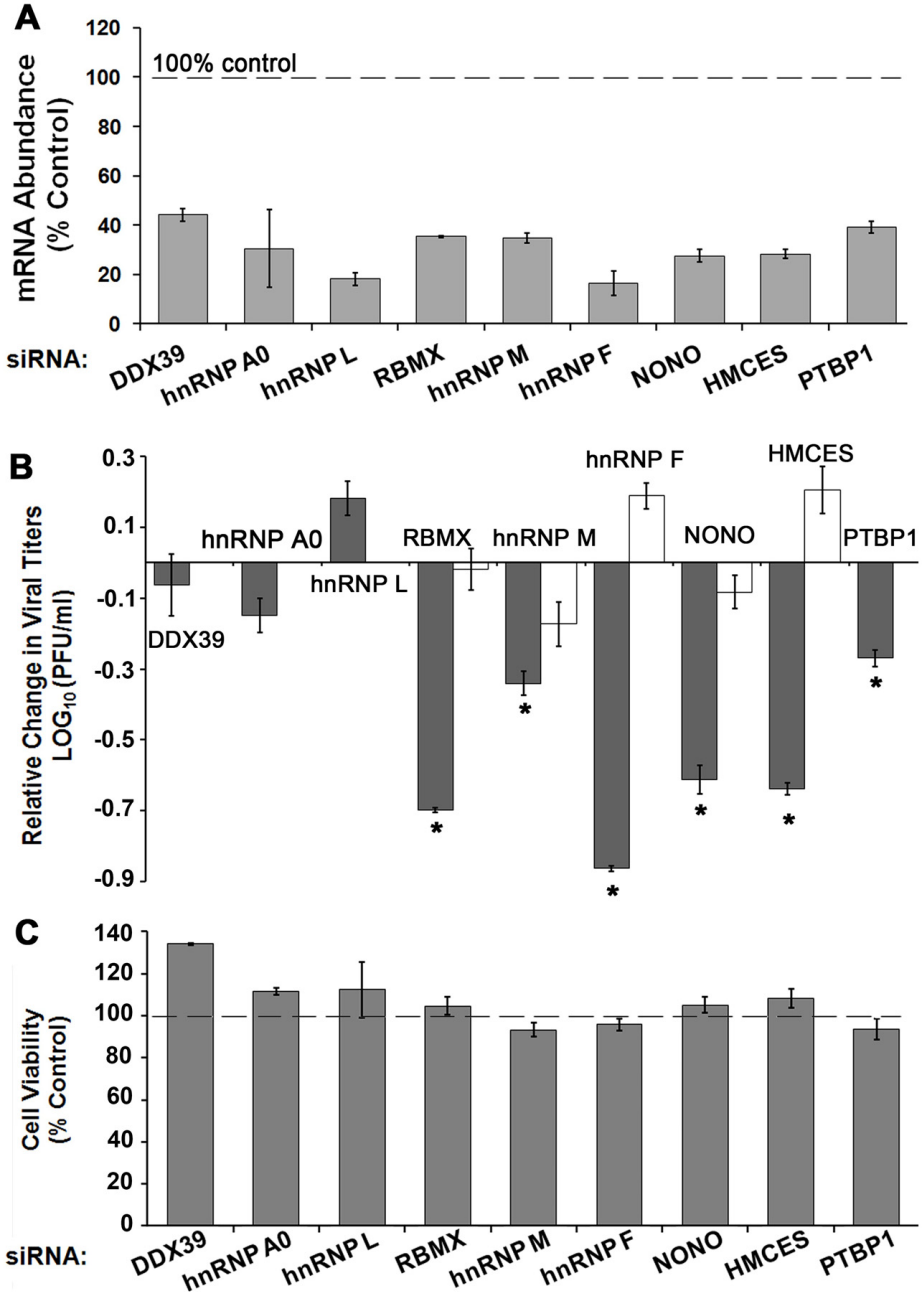
Depletion of cellular proteins identified by qTUX-MS affect DENV titer. HeLa^UPRT^ cells were transfected with either control or specific siRNAs and 48 hours post transfection were infected with DENV2, Ad5 or tested for cell viability (C). 40 hours post infection knockdown efficiencies were assessed at the mRNA levels (A), and DENV and Ad5 titers were determined (B). (**A**) mRNA levels were measured by RT-qPCR and quantified using the 2^-ΔΔCt^ approach and normalized to β-actin mRNA levels. (**B**) DENV and Ad5 were collected at either 40 hpi or 30 hpi, respectively, and titers were determined by plaque assays. Average viral titers for DENV (dark grey) and Ad5 (white) are reported relative to the titer obtained from the control siRNA knockdown (Log10 = 0). Error bars represent ± standard error, p<0.05 (*) are shown. Absolute values for DENV and Ad5 titers are shown in S6 Fig. (**C**) Relative viability of non-infected cells was measured using an MTT assay (Invitrogen) 48 hours following knockdown by the indicated siRNAs.

Since most of the factors were associated with RNA processing in the Reactome analysis (Fig 4), we focused on these factors for functional analysis of their roles in dengue infection. We have randomly selected six qTUX-MS identified host proteins with functions in RNA processing/translation, which were enhanced in the DENV sample ranging from 1.32-to 2.26-fold (DENV/mock). Thus, by validating factors that are only modestly enhanced in the qTUX-MS analysis this will indicate if the qTUX-MS identified factors that are near the cut-off of 1.5-fold are bona fide host factors or not. We assessed the effect of siRNA knockdown of these factors (Fig 5A) on viral production. HeLa^UPRT^ cells were used for siRNA silencing experiments due to higher siRNA transfection efficiency compared with Huh7.5^UPRT^ cells. Knockdown of five out of six qTUX-MS identified candidates: Non-POU domain-containing octamer-binding protein (NONO), Embryonic stem cell-specific 5-hydroxymethylcytosine-binding protein (HMCES), RBMX (RNA-binding motif protein, X chromosome), hnRNP M and hnRNP F significantly decreased DENV production, while knockdown of hnRNP L had no effect on DENV titer (Fig 5B; S5 Fig). Knockdown of PTBP1, a positive control [7, 8], also resulted in decreased viral titers (Fig 5). For negative controls, we selected two RNA binding proteins (DDX39 and hnRNP A0) that were not identified by qTUX-MS. DENV titers were not altered following silencing of these two proteins, suggesting that only specific RNA binding proteins are used by DENV. Altogether, our data suggests that RBMX, NONO, HMCES, hnRNP M, hnRNP F are required for viral production. These results demonstrate that qTUX-MS is a robust method with a low false discovery rate for high-throughput identification of viral host factors.

Reduced viral amplification could be due to compromised cell fitness rather than a specific requirement of the virus for a particular host factor. Using an MTT assay, we confirmed that knockdown of these factors did not impact cell viability (Fig 5C). As a positive control, knockdown of G3BP2 did reduce cell fitness, as previously shown (S6 Fig) [37]. To more rigorously rule out any potential effects of host factor knockdown on cell fitness that would affect viral amplification, an unrelated virus (adenovirus 5), was amplified following knockdowns of the candidate factors. Adenovirus 5 replication was not significantly decreased by RBMX, NONO, hnRNP M, hnRNP F or HMCES siRNA knockdown (Fig 5B and S6C Fig) demonstrating that knockdown of these factors does not affect cell fitness for viral amplification. Given that these proteins bind directly to viral RNA and are required for viral amplification, RBMX, NONO, hnRNP M, hnRNP F and HMCES are novel DENV host factors.

### Assessing the temporal roles of qTUX-MS host factors during DENV infection

**Fig 6:**
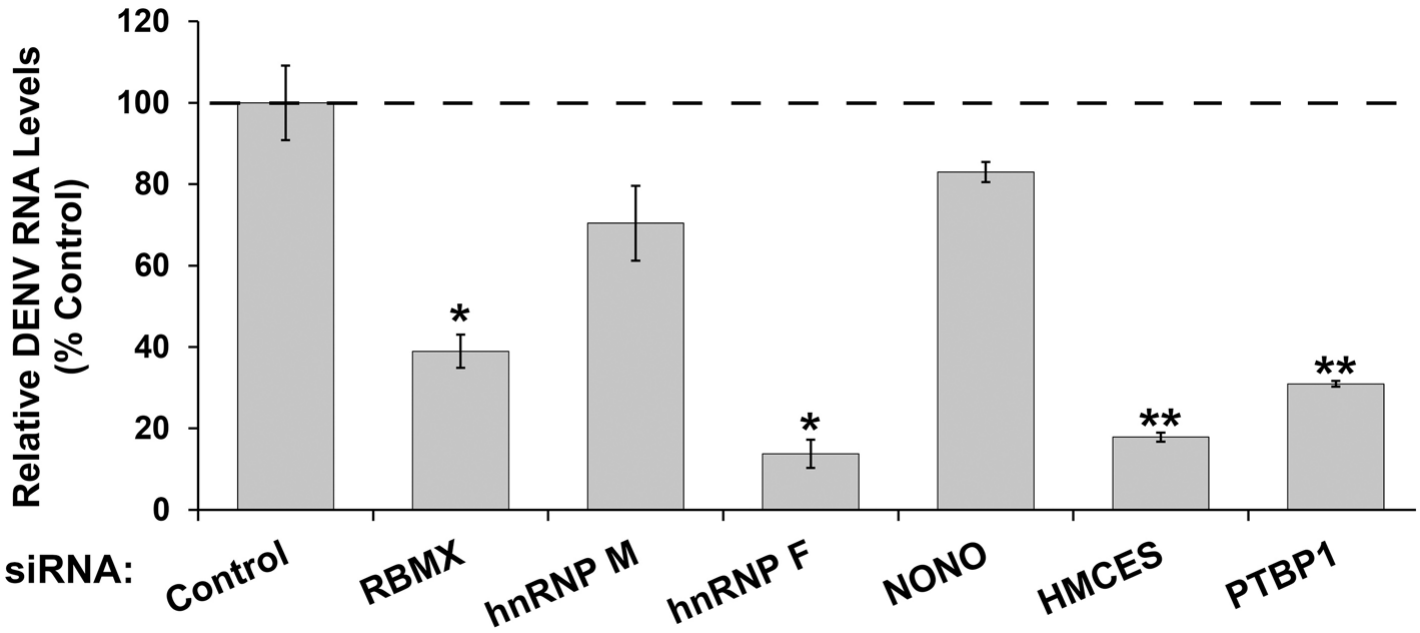
Knockdown of qTUX-MS host factors reduces DENV RNA levels. Cells were infected with DENV 48 hours following siRNA transfections and collected 40 hpi. Total RNA was extracted from the cells and viral RNA levels were determined using qRT-PCR analysis. Each bar represents an average value of the viral RNA normalized to β-actin mRNA levels from at three independent experiments in the indicated host factor depleted cells relative to control siRNA knockdown. Error bars represent ± standard deviation, p<0.05 (*), p<0.01 (**) are shown.

To determine if the host factor is required for a step prior or subsequent to viral replication, vRNA was quantified by qRT-PCR in the DENV infected cells knocked down for RBMX, NONO, hnRNP M, hnRNP F or HMCES (Fig 6). As a positive control, knockdown of PTBP1, which is required for DENV replication [7], reduced DENV RNA levels. Similarly, the intracellular vRNA levels were reduced in cells knocked down for either hnRNP F, RBMX or HMCES. The decrease in dengue RNA levels (Fig 6) is consistent with the decrease in viral titers (Fig 5B). Thus, hnRNP F, RBMX or HMCES are required for the early steps in the viral replication cycle, such as translation, replication or RNA stability. In contrast, knockdown of hnRNP M and NONO did not change intracellular viral RNA levels despite the dramatic decrease in viral titers, suggesting they may play a role downstream of replication. Altogether, we have identified and validated five novel host factors for DENV, demonstrating that qTUX-MS can identify factors that function at different stages of the virus life cycle.

## Discussion

An estimated 40% of the world’s population is at risk from dengue for which vaccines or antivirals are not yet available. Since diagnostic tests can detect DENV infection at early stages, administration of antivirals could significantly improve survival rates as viral load is correlated with symptom severity [38]. Using antivirals that target host factors may limit the appearance of drug-resistant viruses and may be effective for all DENV serotypes and possibly for multiple flaviviruses [5, 6, 39, 40]. The qTUX-MS analysis identified 79 novel cellular proteins, for which the majority are distinct from those previously identified for poliovirus using a similar approach [15]. This suggests that unrelated RNA viruses have evolved to utilize distinct host RNA binding proteins. Interestingly, PTBP1 and NONO, which were identified in both the poliovirus and DENV TUX-MS analyses, were shown to be required for production of both viruses (this study, Figs 5 and 6) [15, 41, 42]. Further analysis of virus-specific and shared host factors will reveal whether unrelated viruses utilize similar or diverse mechanisms to control viral RNA replication, processing and packaging within cells. The host factors that enhance amplification of both poliovirus and dengue, (Fig 2B) could serve as attractive targets for the development of broad-spectrum antivirals.

The novel DENV RNA interactions identified in our study reveal a large network of cellular proteins which belong to different functional classes primarily associated with the nucleic acid metabolism, including numerous components of splicing, RNA processing and translation machineries. These factors likely play direct roles in DENV translation, replication or packaging. In addition, we have detected multiple components of cell signaling and stress response, such as several members of 14-3-3 adapter proteins, heat shock proteins and β-catenin. These factors are known to regulate diverse pathways, including host innate immune and cellular homeostasis [43–45] suggesting their possible role in host antiviral response or viral strategies to subvert the innate immune response.

We demonstrated that the majority of the qTUX-MS factors that we selected for validation were required for efficient DENV amplification (Fig 5). Specifically, we found that hnRNP F, HMCES and RBMX are required for the early steps in the viral life cycle. In contrast, hnRNP M and NONO appear to act downstream of viral RNA replication (Fig 6), which may be significant given that both have been shown to be in a complex together [46]. NONO and its binding partners are predominantly nuclear, bind RNA, and are involved in pre-mRNA processing, splicing, and RNA transport, as well as in transcriptional activation and repression [47–49]. Interestingly, other NONO binding partners: PSF/SFPQ (polypyrimidine tract-binding protein (PTB)-associated splicing factor) and Matrin3 were identified by qTUX-MS as well.

Many of the qTUX-MS identified cellular proteins are hnRNPs, which encompass a large class of RNA binding proteins that either localize to the nucleus or shuttle between the nucleus and the cytoplasm in order to perform multiple functions in RNA metabolism, from transcription to RNA turnover [50–52]. Importantly, the vast majority of these factors have established roles in viral infections or in modulating the antiviral host response to various viruses [53–56], including DENV (S2 Table and references within). Our study establishes that RBMX (hnRNP G), hnRNP F and hnRNP M are required for efficient DENV amplification (Fig 5B). Since several hnRNPs, such as PTBP1 (hnRNP I), hnRNP A1 and hnRNP K, were previously shown to re-localize from the nucleus to the viral replication sites during DENV infection [8, 57, 58], RBMX, hnRNP F, hnRNP M and possibly other nuclear qTUX-MS identified factors are also likely to be either actively recruited to the viral replication sites or retained in the cytoplasm upon DENV infection. Interestingly, the qTUX-MS identified hnRNPs affect different steps in the DENV life cycle (Fig 6), suggesting that they have distinct functions during infection. This is consistent with studies that have suggested that DENV RNA structures are dynamic during the viral life cycle [59] and may suggest that host factors play an important role in these structural changes. Furthermore, we showed that knockdown of some hnRNPs (hnRNP A0 and hnRNP L) did not affect dengue viral titers significantly demonstrating that only certain hnRNPs are required for DENV amplification. Since some of hnRNPs are known to modulate cellular gene expression in response to dengue infection [60, 61], we can not rule out that some of the observed effects on virus titers derive from their roles in regulating host mRNAs.

Among the numerous qTUX-MS identified factors of interest, our study is the first to demonstrate the involvement of HMCES (or C3orf37) in viral infection. While the cellular role of human HMCES is currently unknown, the mouse homologue was suggested to be an RNA-binding protein and predicted to contain a putative peptidase domain [62, 63]. Interestingly, it is possible that the nucleic acid binding domain enhances the protease activity or visa versa as has been shown for other such proteins [64–66]. For example, Adenovirus uses a nucleic acid binding protease to localize the protease activity to the viral substrates [64]. It has been suggested that the protease is recruited to the empty capsid as an inactive protease, then it becomes fully activated once bound to the viral DNA inside the virion. Using the DNA as a guide wire, it moves along the nucleic acid, searching for capsid and core proteins to cleave, which is required to render the viral particle infectious [64, 67, 68]. However, since we observed that knockdown of HMCES resulted in a decrease in viral RNA, it seems more likely that it might participate in translation, replication or the switch from translation to replication as has been shown for other nucleic acid binding proteases [65, 69].

Only one of the qTUX-MS identified factors was increased at the protein level in whole cells following DENV infection, suggesting that qTUX-MS identifications derived from the specific associations to vRNA rather than changes in protein abundances. Our analysis of the host cell proteome upon infection also revealed interesting changes, including up regulation of proteins in the ubiquitin pathway and down regulation of transporter proteins. The ubiquitin-proteasome pathway is one of two major cellular pathways used to degrade 80 to 90% of proteins. Previous studies on DENV infected cell lines and patient samples showed that the ubiquitin pathway was upregulated [31, 39, 70]. Many groups have consistently shown that the ubiquitin proteasome pathway is critical for amplification of a number of flaviruses, including DENV and West Nile virus [31, 34, 71, 72]. However, it remains controversial as to which step in viral amplification is affected by ubiquitination, but it appears to be early during internalization or viral genome release [33, 39, 73]. Further studies will be required to understand how DENV up-regulates the pathway and the mechanism that ubiquitination has in DENV amplification.

Altogether, our study has significantly increased the number of cellular proteins known to interact with the DENV RNA during a live infection in cells. We have also placed these interactions in the context of proteome abundance changes in the infected cells. A recent study by Phillips and colleagues [13] exploited a cross-linking label-free MS approach to identify DENV RNA associating proteins in cell culture by cross-linking the RNA to the proteins using short wavelength UV light and isolating DENV RNA bound proteins by anti-sense DNA affinity capture [74]. While their method identified several DENV host factors [12, 75–77], the qTUX-MS method reported here resulted in improved identification of known dengue host factors and putative DENV RNA interacting proteins. There could be several reasons for these results, such as, the qTUX-MS approach achieves greater cross-linking efficiency by using long wave UV light to form crosslinks to 4-thio-uridines compared to short-wavelength UV light, which is inherently inefficient [78]. Moreover, since thio-uridine is a zero distance cross-linker for RNA-bound proteins at long UV wavelengths and protein-protein cross-links are not formed at long UV wavelengths, qTUX-MS may also have achieved improved specificity, as only proteins in direct contact with the viral RNA would be captured [23]. Additionally, qTUX-MS used an MS-based SILAC approach to determine which host proteins were specifically enriched in the vRNA isolations versus mock. Though isotope-labelling is not applicable in all model systems, it does afford greater quantitative accuracy compared to label-free MS strategies [16].

Overall, the qTUX-MS method identified 93 cellular proteins that bind to DENV RNA, which include 14 previously known or putative interactions. Importantly, five out of the six qTUX-MS identified novel factors that were tested were shown to be bona fide host factors. We used robust assays to show that the identified host factors were specifically required for DENV amplification and did not merely result in a decrease in cell fitness for viral amplification. Future studies will reveal whether the identified factors may also be required for other *flavivirus* infections that cause life-threatening illnesses, such as yellow fever, West Nile, Zika, Japanese and tick-borne encephalitis. Therefore, our data demonstrates that qTUX-MS is an effective technique for identifying novel virus host factors that can be used for a broad spectrum of RNA viruses by simply designing antisense DNA oligonucleotides to allow for efficient sequence-specific isolation of the vRNA.

## Supporting Information

S1 Table The list of the primers and siRNA used in this study. (DOCX)

S2 Table Known and putative DENV host factors/interacting proteins identified by qTUX-MS. ^a^UniProt accession number, ^b^DENV/Mock enrichment ratio calculated by MaxQuant, ^c^Number of peptides used to calculate the DENV/Mock TUX-MS ratio, ^d^The variance in the DENV/Mock TUX-MS ratio, calculated as the coefficient of variation in peptide ratio counts.
(DOCX)

S1 Methods Text file with detailed description of the indicated methods. (DOC)

S1 Dataset Excel spreadsheet of qTUX-MS quantified proteins and of SILAC quantified proteins from whole cell analyses. (**A**). Table containing all proteins quantified by qTUX-MS, including proteins enriched in DENV infection (red highlighted rows) and those that did not meet the specificity threshold. For each protein group, the following are provided (from left to right), UniProt Accession number, gene name, protein name, the linear and Log2 DENV/Mock qTUX-MS enrichment ratios (columns D and E), the qTUX-MS ratio variability, the number of qTUX-MS quantified peptides, the average Log2 DENV/Mock whole cell relative abundance SILAC ratio, whether this ratio was up (U) or down (D) regulated by more than ± 1.75-fold, the average SILAC ratio variability, the average number of quantified peptides, the number of razor+unique peptides and sequence coverage for qTUX-MS, the protein’s molecular weight and sequence length, and the complete FASTA header entry for the primary protein group member. Columns H–K are cross-referenced from the respective whole cell data (B). ND = not detected. (**B**) Table containing all proteins quantified in whole cell lysates by SILAC. For each protein group, the following are provided (from left to right), UniProt Accession number, gene name, protein name, the Log2 DENV/Mock relative abundance ratios for replicates 1, 2, and the average (columns D and E), whether this ratio was up (U) or down (D) regulated by more than ± 1.75-fold. For each replicate, the following are provide: the SILAC ratio variability, the number of quantified peptides, the number of razor+unique peptides, and the number of unique peptides, total protein intensity for light (DENV) and heavy (MOCK) conditions, and the overall sequence coverage. The complete FASTA header entry is listed for the primary protein group accession number.
(XLSX)

S1 Fig The relative abundances for the qTUX-MS identified factors. The relative log_2_ abundance of each protein (indicated by its gene symbol) was compared for the qTUX-MS (grey bars) and whole cell analysis (white bars) for (A) known DENV pro- and anti-viral factors, (B) known DENV vRNA-binding proteins, and (C) qTUX-MS identified putative factors. The error bars represent the protein ratio variability, calculated from individual light/heavy peptide ratios. Dashed line indicates the 1.5-fold enrichment threshold.
(TIF)

S2 Fig PANTHER gene enrichment analysis of proteins quantified from Huh7.5 whole cell lysates. The genes and relative expression ratios of host proteins quantified from Huh7.5 whole cell lysates (N = 4907) were submitted to PANTHER gene enrichment using the default settings (www.pantherdb.org). The enrichment of gene functions was performed using the PANTHER “Pathways” and “Protein Class” ontologies. Only the statistically significant ontology terms are displayed, along with the number of genes annotated in that ontology, whether that set of genes was systematically up (+) or down (-) regulated, and the corresponding p-value.
(TIF)

S3 Fig Protein network associated with the Ubiquitin Pathway PANTHER protein class clustered by Reactome relationships (top) and overlaid with log_2_ DENV/Mock relative abundance changes (bottom). PANTHER gene enrichment annotated 44 proteins to the Ubiquitin-Proteasome Pathway, which on average were significantly up-regulated upon DENV infection. The corresponding genes were analyzed by the Reactome Functional Interaction (FI) Cytoscape plug-in. Sub-networks (≥ 2 genes per network) were assembled with nodes colors representing functional clustering by Reactome FI (*top*) or log_2_ DENV/Mock relative abundance changes (*bottom*). Network edges represent Reactome functional interactions:–, protein complex; →, activating;–|, inhibiting; —, predicted. Clusters were labeled with protein functions/activities representative of the majority of proteins within each cluster.
(TIF)

S4 Fig Protein network associated with the transporter PANTHER protein class clustered by Reactome relationships. PANTHER gene enrichment classified 209 proteins annotated as transporters, which on average were significantly down regulated upon DENV infection. The corresponding genes were analyzed by the Reactome Functional Interaction (FI) Cytoscape plug-in. Four distinct sub-networks (≥ 3 genes per network) were assembled containing 71 out of the 209 genes. Nodes colors represent functional clustering by Reactome FI. Network edges represent Reactome functional interactions:–, protein complex; →, activating;–|, inhibiting; —, predicted. Clusters were labelled with the transporter classes and/or activities represented by the majority of proteins within that cluster.
(TIF)

S5 Fig Protein network associated with the RNA protein binding PANTHER protein class clustered by Reactome relationships (*top*) and overlaid with log2 DENV/Mock relative abundance changes (*bottom*). PANTHER gene enrichment classified 523 proteins annotated as RNA binding proteins, which on average were significantly down regulated upon DENV infection. The corresponding genes were analyzed by the Reactome Functional Interaction (FI) Cytoscape plug-in. Six sub-networks (≥ 3 genes per network) were assembled with modes colors representing functional clustering by Reactome FI (top) or log2 DENV/Mock relative abundance changes (bottom). Network edges represent Reactome functional interactions:–, protein complex; →, activating;–|, inhibiting; —, predicted. Clusters were labelled with the transporter classes and/or activities represented by the majority of proteins within that cluster.
(TIF)

S6 Fig siRNA knockdown of cellular proteins identified by qTUX-MS affect DENV titer. **(A)** HeLa^UPRT^ cells were transfected with either control or specific siRNAs. 24 hours post transfection cells were counted and seeded (4 X 10^5^ cells/well) in 6-well plates in triplicates. 48 hours post transfection cells were infected with DENV2 at MOI 0.1 and virus released to the media collected 40 hours post infection. DENV2 titers were measured using plaque assays. The bars represent average values from triplicate, a standard error is reported. The representative data from one of at least two independent experiments is shown. (**B**) To verify sensitivity of the MTT assay, we performed siRNA knockdown of G3BP2, which is known to bind DENV and was previously shown to affect cell viability [37, 75]. Relative viability of non-infected cells was measured using an MTT assay (Invitrogen) and represented exactly as described in Fig 5C. Cell viability or proliferation is decreased by knockdown of G3BP2 compared with control siRNA (p<0.01). siRNA transfection was performed as described in the methods section using previously published siRNA sequence [37]. In cells treated with G3BP2-specific siRNA but not control siRNA, G3BP2 protein was knocked down to the levels undetectable by western analysis using antibodies against G3BP2 (Abcam, ab86135). (C). HeLa^UPRT^ cells were seeded 4.0 X 10^5^ cells in 60 mm plates and transfected with either control or specific siRNAs. 24 hours post transfection cells were infected with Ad5 at MOI 0.1 and collected 30 hours post infection. siRNA knockdown efficiency was determined by measuring respective mRNA levels in comparison to β-actin mRNA abundance as described in the Experimental Section and reported relative to control siRNA transfection (upper panel). Ad5 titers were determined using plaque assays on 911 cells. The bars represent average values from triplicate, a standard error is reported. The representative data from one of at least three independent experiments is shown.
(TIF)
