# Supplementary material for: Identification of RNA Binding Proteins Associated with Dengue Virus RNA in Infected Cells Reveals Temporally Distinct Host Factor Requirements
Source: PLoS Negl Trop Dis. 2016 Aug 24;10(8):e0004921. doi: 10.1371/journal.pntd.0004921 (PMC4996428; doi:10.1371/journal.pntd.0004921)
Supplement: S1 Table — (DOCX) [file pntd.0004921.s001.docx]

**Supplementary Table 1. The list of primers and siRNA used in the study.**

The list of the primers used to generate the antisense DNA for binding to the beads.

| **Oligo** | | | **Sequence** |
| --- | --- | --- | --- |
| DENV2 4914 (reverse) | | | 5’ (biotin) ggggaaaagtccagagatacg 3’ |
| DENV2 4350 (forward) | | | 5’ cctgtcaataacaatatcagaagatgg 3’ |
| DENV2 4740 (forward) | | | 5’ gaaagacctaatatcatatggagg 3’ |
| The list of siRNA used in the study. | | | |
| **Gene** | **Source** | **Sequence (5’ to 3’)** | |
| hnRNPM | IDT | \| UAUGCUUGUUUAGGACUUC  GAAGUCCUAAACAAGCAUA \| \| --- \| | |
| hnRNPL | IDT | \| AAAGGAAAGAGAAAUGUCTT  GACAUUUCUCUUUCCUUUTT \| \| --- \| | |
| hnRNPA0 | Ambion | GACUUUACGUGUUAAUUCUtt  AGAAUUAACACGUAAAGUCtt | |
| NONO | Ambion | GGCUUGACUAUUGACCUGATT  UCAGGUCAAUAGUCAAGCCTT | |
| DDX39 | Ambion | GAGUUUAACCAGGUGAUAAtt  UUAUCACCUGGUUAAACUCca | |
| RBMX | IDT | \| UCAAGAGGAUAUAGCGAUTT  AUCGCUAUAUCCUCUUGATT \| \| --- \| | |
| hnRNPF | Ambion | GAAUGUAUGACCACAGAUAtt  UAUCUGUGGUCAUACAUUCcg | |
| HMCES | Ambion | UCGACUUGGUGGUCAAAAAtt  UUUUUGACCACCAAGUCGAca | |
| PTBP1 | IDT | \| AACUUCCAUCAUUCCAGAAA  UUCUCUGGAAUGAUGGAAGUU \| \| --- \| | |

| The list of the qPCR primers. | |  |
| --- | --- | --- |
| **primer** | **sequence (5’ to 3’)** | **PCR efficiency (%)** |
| DENV for | AGTTGTTAGTCTACGTGGACCGAC | 101 |
| DENV rev | CGCGTTTCAGCATATTGAAAGG |  |
| ACTB for | GGGCTCTTCCAGCCTTCC | 92 |
| ACTB rev | TGTCCAGGTCACACTTGATG |  |
| HNRNPL for | TTCTGCTTATATGGCAATGTGG | 90 |
| HNRNPL rev | GACTGACCAGGCATGATGG |  |
| HNRNPM for | CTCTTAATGGACGCTGAAGGAAA | 110 |
| HNRNPM rev | CGCTCAGACTATGCTTGTTTAGG |  |
| RBMX for | TGGAAGCAGTCGCTATGATG | 104 |
| RBMX rev | GAGGGTACCCCCTTTCCATA |  |
| HNRNPF for | CTCAGTGATGGCTACGGCTT | 100 |
| HNRNPF rev | CTCACTGTCGCCGTATCTGT |  |
| HNRNPA0 for | AGGATCCATTCCGTACCGTT | 97 |
| HNRNPA0 rev | AAGCCACATCCACCACTTCA |  |
| DDX39 for | CTGGGGCTCTTCCTCTTCAT | 109 |
| DDX39 rev | AGCAACTCGTGTCTGAGCG |  |
| HMCES for | GTGGTGAACAACTCGCGAAA | 94 |
| HMCES rev | CTTGCCCTGAGCTCCTTTTTGA |  |
| PTBP1 for | AGCGCGTGAAGATCCTGTTC | 90 |
| PTBP1 rev | CAGGGGTGAGTTGCCGTAG |  |
| NONO for | GGCAGGCGAAGTCTTCATTCA | 107 |
| NONO rev | TGGCAATCTCCGCTAGGGT |  |
