# Supplementary material for: Identification of RNA Binding Proteins Associated with Dengue Virus RNA in Infected Cells Reveals Temporally Distinct Host Factor Requirements
Source: PLoS Negl Trop Dis. 2016 Aug 24;10(8):e0004921. doi: 10.1371/journal.pntd.0004921 (PMC4996428; doi:10.1371/journal.pntd.0004921)
Supplement: S2 Table — aUniProt accession number, bDENV/Mock enrichment ratio calculated by MaxQuant, cNumber of peptides used to calculate the DENV/Mock TUX-MS ratio, dThe variance in the DENV/Mock TUX-MS ratio, calculated as the coefficient of variation in peptide ratio counts. (DOCX) [file pntd.0004921.s002.docx]

**Supplementary Table 2. Known and putative DENV host factors/interacting proteins identified by qTUX-MS.**

| **Gene** | **Acc. No.^a^** | **Protein name** | **Unique peptides** | **qTUX-MS Ratio^b^** | **Ratio count^c^** | **Ratio variance [%]^d^** | **Primary Host Cell Function** | **Function in DENV** | Ref |
| --- | --- | --- | --- | --- | --- | --- | --- | --- | --- |
| **Known DENV Proviral and Antiviral Factors** | | | | | | | | | |
| **CALR** | P27797 | Calreticulin | 9 | 4.51 | 13 | 37.1 | Protein Folding | binds DENV 3'UTR, required for efficient virus production | (1,2) |
| **CANX** | P27824 | Calnexin | 3 | 4.33 | 3 | 19.3 | Protein Folding | binds protein E; required for efficient virus production | (3) |
| **ILF3** | Q12906 | Interleukin enhancer-binding factor 3 | 3 | 1.81 | 4 | 37.2 | RNA | binds DENV 3'UTR, required for efficient virus production | (4) |
| **PTBP1** | P26599 | Polypyrimidine tract-binding protein 1 | 3 | 1.79 | 3 | 17.8 | RNA | binds DENV 3'UTR, required for efficient viral replication | (5-7) |
| **HNRNPH1** | P31943 | Heterogeneous nuclear ribonucleoprotein H | 10 | 1.41 | 25 | 27.6 | RNA | binds DENV 3’UTR, required for DENV multiplication | (8,9) |
| **HNRNPK** | P61978 | Heterogeneous nuclear ribonucleoprotein K | 10 | 1.35 | 19 | 32.9 | RNA | interacts with core protein, required for DENV multiplication | (10,11) |
| **EIF4A1** | P60842 | Eukaryotic initiation factor 4A-I | 4 | 1.25 | 3 | 36.9 | Translation | binds DENV 3’UTR, binds NS4A, has antiviral activity | (5,12) |
| **Cellular Factors Identified by qTUX-MS and Other Approaches Through Interactions with DENV** | | | | | | | | | |
| **WDR12** | Q9GZL7 | Ribosome biogenesis protein WDR12 | 3 | 3.83 | 3 | 34.6 | RNA | binds DENV 3'UTR | (13) |
| **ILF2** | Q12905 | Interleukin enhancer-binding factor 2 | 3 | 3.17 | 4 | 16.8 | RNA | binds DENV 3'UTR | (4) |
| **PRPF19** | Q9UMS4 | Pre-mRNA-processing factor 19 | 3 | 2.62 | 3 | 21.1 | RNA | binds DENV 3'UTR | (13) |
| **HSPB1** | P04792 | Heat shock protein beta-1 | 10 | 2.32 | 29 | 40.7 | Protein Folding | binds DENV 3'UTR | (13) |
| **SSBP1** | Q04837 | Single-stranded DNA-binding protein, mitochondrial | 9 | 2.17 | 49 | 40.4 | DNA/  Transcription | binds DENV RNA | (14) |
| **CLU** | P10909 | Clusterin | 3 | 2.02 | 6 | 4.5 | Other | binds NS1 | (15) |
| **DHX9** | Q08211 | ATP-dependent RNA helicase A | 4 | 1.72 | 3 | 21.5 | RNA | binds DENV 3'UTR | (4) |
| **STRAP** | Q9Y3F4 | Serine-threonine kinase receptor-associated protein | 4 | 1.74 | 4 | 29.1 | RNA | binds DENV 3'UTR | (13) |
| **HNRNPF** | P52597 | Heterogeneous nuclear ribonucleoprotein F | 5 | 1.84 | 11 | 26.7 | RNA | binds DENV 3'UTR | (13) |
| **COPE** | O14579 | Coatomer subunit epsilon | 6 | 1.70 | 7 | 20.2 | Cytoskeleton | binds DENV 3'UTR | (13) |
| **RBMX** | P38159 | RNA-binding motif protein, X chromosome | 2 | 1.32 | 4 | 8.6 | RNA | binds DENV 3'UTR | (13,16) |
| **HNRNPA1** | P09651 | Heterogeneous nuclear ribonucleoprotein A1 | 8 | 1.27 | 20 | 26.5 | RNA | binds DENV 3'UTR | (9,13) |

**Putative DENV host factor candidates identified by qTUX-MS**

| **Gene** | **Acc. No.^a^** | **Protein name** | **Unique peptides** | **qTUX-MS Ratio^b^** | **Ratio count^c^** | **Ratio variance [%]^d^** | **Primary Host Cell Function** |
| --- | --- | --- | --- | --- | --- | --- | --- |
| **RNH1** | P13489 | Ribonuclease inhibitor | 18 | 46.57 | 47 | 175.0 | Other/Unknown |
| **YWHAB** | P31946 | 14-3-3 protein beta/alpha | 5 | 7.15 | 4 | 14.9 | Signal Transduction |
| **POLR2C** | P19387 | DNA-directed RNA polymerase II subunit RPB3 | 3 | 5.39 | 3 | 32.9 | RNA |
| **LMNB2** | Q03252 | Lamin-B2 | 4 | 5.36 | 3 | 16.0 | Other/Unknown |
| **TUBB6** | Q9BUF5 | Tubulin beta-6 chain | 5 | 4.87 | 20 | 25.3 | Cytoskeleton |
| **LMNA** | P02545 | Prelamin-A/C;Lamin-A/C | 9 | 4.36 | 5 | 56.2 | Other/Unknown |
| **RPS27A** | P62979 | Ubiquitin-40S ribosomal protein S27a | 3 | 4.28 | 10 | 26.9 | RNA |
| **SLC3A2** | P08195 | 4F2 cell-surface antigen heavy chain | 5 | 4.14 | 6 | 15.3 | Cytoskeleton |
| **TUBB3** | Q13509 | Tubulin beta-3 chain | 5 | 4.09 | 13 | 22.8 | Cytoskeleton |
| **PUF60** | Q9UHX1 | Poly(U)-binding-splicing factor PUF60 | 4 | 3.93 | 10 | 18.2 | RNA |
| **YWHAZ** | P63104 | 14-3-3 protein zeta/delta | 4 | 3.79 | 4 | 12.8 | Signal Transduction |
| **PCNA** | P12004 | Proliferating cell nuclear antigen | 4 | 3.76 | 6 | 18.5 | DNA/Transcription |
| **EIF6** | P56537 | Eukaryotic translation initiation factor 6 | 4 | 3.76 | 8 | 18.5 | RNA |
| **ERH** | P84090 | Enhancer of rudimentary homolog | 3 | 3.50 | 8 | 19.4 | Other/Unknown |
| **RAB1B** | Q9H0U4 | Ras-related protein Rab-1B | 2 | 3.39 | 4 | 41.3 | Cytoskeleton |
| **GRWD1** | Q9BQ67 | Glutamate-rich WD repeat-containing protein 1 | 3 | 3.29 | 3 | 22.6 | Other/Unknown |
| **TGM3** | Q08188 | Protein-glutamine gamma-glutamyltransferase E | 8 | 3.28 | 6 | 115.0 | Other/Unknown |
| **EEF1D** | P29692 | Elongation factor 1-delta | 3 | 3.24 | 7 | 27.3 | RNA |
| **TUBB2B** | Q9BVA1 | Tubulin beta-2B chain | 2 | 3.09 | 8 | 23.1 | Cytoskeleton |
| **YWHAG** | P61981 | 14-3-3 protein gamma | 3 | 3.06 | 4 | 95.3 | Signal Transduction |
| **NAP1L1** | P55209 | Nucleosome assembly protein 1-like 1 | 2 | 2.99 | 6 | 37.0 | DNA/Transcription |
| **YWHAE** | P62258 | 14-3-3 protein epsilon | 3 | 2.98 | 4 | 24.7 | Signal Transduction |
| **GGCT** | O75223 | Gamma-glutamylcyclotransferase | 4 | 2.92 | 3 | 148.8 | Other/Unknown |
| **SF3B5** | Q9BWJ5 | Splicing factor 3B subunit 5 | 1 | 2.89 | 3 | 24.0 | RNA |
| **CASP14** | P31944 | Caspase-14 | 6 | 2.83 | 10 | 105.7 | Other/Unknown |
| **TUBB4B** | P68371 | Tubulin beta-4B chain | 21 | 2.74 | 100 | 26.0 | Cytoskeleton |
| **BANF1** | O75531 | Barrier-to-autointegration factor | 4 | 2.73 | 4 | 23.2 | DNA/Transcription |
| **FIP1L1** | Q6UN15 | Pre-mRNA 3-end-processing factor FIP1 | 2 | 2.70 | 3 | 59.1 | RNA |
| **SERPINB3** | P29508 | Serpin B3 | 6 | 2.64 | 7 | 166.9 | Signal Transduction |
| **TUBB** | P07437 | Tubulin beta chain | 4 | 2.54 | 29 | 17.7 | Cytoskeleton |
| **SF3B3** | Q15393 | Splicing factor 3B subunit 3 | 19 | 2.50 | 53 | 25.1 | RNA |
| **BCAS2** | O75934 | Pre-mRNA-splicing factor SPF27 | 3 | 2.49 | 4 | 32.1 | RNA |
| **CAT** | P04040 | Catalase | 3 | 2.43 | 3 | 115.9 | Other/Unknown |
| **ATP5B** | P06576 | ATP synthase subunit beta, mitochondrial | 5 | 2.41 | 8 | 28.7 | Other/Unknown |
| **CHERP** | Q8IWX8 | Calcium homeostasis endoplasmic reticulum protein | 5 | 2.38 | 5 | 28.4 | Other/Unknown |
| **POLR2H** | P52434 | DNA-directed RNA polymerases I, II, and III subunit RPABC3 | 3 | 2.38 | 4 | 24.7 | RNA |
| **C1QBP** | Q07021 | Complement component 1 Q subcomponent-binding protein, mitochondrial | 3 | 2.36 | 3 | 32.4 | Signal Transduction |
| **NPM1** | P06748 | Nucleophosmin | 7 | 2.29 | 20 | 39.9 | RNA |
| **PAF1** | Q8N7H5 | RNA polymerase II-associated factor 1 homolog | 3 | 2.27 | 3 | 35.5 | RNA |
| **HMCES** | Q96FZ2 | Embryonic stem cell-specific 5-hydroxymethylcytosine-binding protein | 9 | 2.26 | 17 | 46.2 | RNA |
| **EIF4A3** | P38919 | Eukaryotic initiation factor 4A-III | 3 | 2.25 | 3 | 6.4 | RNA |
| **RBBP4** | Q09028 | Histone-binding protein RBBP4 | 8 | 2.22 | 15 | 22.7 | DNA/Transcription |
| **PSMA5** | P28066 | Proteasome subunit alpha type-5 | 3 | 2.19 | 3 | 50.7 | Other/Unknown |
| **HSP90AB1** | P08238 | Heat shock protein HSP 90-beta | 6 | 2.10 | 14 | 27.1 | Protein Folding |
| **RBBP7** | Q16576 | Histone-binding protein RBBP7 | 4 | 2.09 | 3 | 29.4 | DNA/Transcription |
| **SLC1A5** | Q15758 | Neutral amino acid transporter B(0) | 1 | 1.99 | 3 | 48.2 | Cytoskeleton |
| **HSPA5** | P11021 | 78 kDa glucose-regulated protein | 7 | 1.98 | 10 | 50.3 | Protein Folding |
| **TUBA1B** | P68363 | Tubulin alpha-1B chain | 18 | 1.98 | 49 | 29.4 | Cytoskeleton |
| **HSP90AA1** | P07900 | Heat shock protein HSP 90-alpha | 3 | 1.97 | 4 | 28.5 | Protein Folding |
| **CBX1** | P83916 | Chromobox protein homolog 1 | 2 | 1.93 | 3 | 26.3 | DNA/Transcription |
| **TXN** | P10599 | Thioredoxin | 3 | 1.92 | 3 | 14.3 | Protein Folding |
| **HSP90B1** | P14625 | Endoplasmin | 4 | 1.92 | 6 | 30.1 | Protein Folding |
| **PCBP1** | Q15365 | Poly(rC)-binding protein 1 | 5 | 1.91 | 11 | 36.2 | RNA |
| **CBX3** | Q13185 | Chromobox protein homolog 3 | 4 | 1.91 | 4 | 11.1 | DNA/Transcription |
| **MAGOH** | P61326 | Protein mago nashi homolog | 3 | 1.90 | 8 | 23.7 | RNA |
| **HNRNPM** | P52272 | Heterogeneous nuclear ribonucleoprotein M | 9 | 1.89 | 9 | 39.8 | RNA |
| **EIF3I** | Q13347 | Eukaryotic translation initiation factor 3 subunit I | 5 | 1.88 | 4 | 27.9 | RNA |
| **TUBA1C** | Q9BQE3 | Tubulin alpha-1C chain | 2 | 1.85 | 3 | 6.0 | Cytoskeleton |
| **SNRPF** | P62306 | Small nuclear ribonucleoprotein F | 2 | 1.85 | 3 | 7.6 | RNA |
| **TOMM22** | Q9NS69 | Mitochondrial import receptor subunit TOM22 homolog | 3 | 1.84 | 3 | 9.3 | Other/Unknown |
| **NONO** | Q15233 | Non-POU domain-containing octamer-binding protein | 2 | 1.84 | 3 | 26.8 | RNA |
| **PSMC4** | P43686 | 26S protease regulatory subunit 6B | 2 | 1.82 | 3 | 32.2 | Other/Unknown |
| **CTSZ** | Q9UBR2 | Cathepsin Z | 5 | 1.81 | 5 | 18.8 | Other/Unknown |
| **EFTUD2** | Q15029 | 116 kDa U5 small nuclear ribonucleoprotein component | 16 | 1.80 | 25 | 25.6 | RNA |
| **ZCCHC8** | Q6NZY4 | Zinc finger CCHC domain-containing protein 8 | 5 | 1.79 | 7 | 27.7 | RNA |
| **FUBP1** | Q96AE4 | Far upstream element-binding protein 1 | 2 | 1.78 | 4 | 43.3 | RNA |
| **SFPQ** | P23246 | Splicing factor, proline- and glutamine-rich | 3 | 1.76 | 7 | 18.9 | RNA |
| **KHSRP** | Q92945 | Far upstream element-binding protein 2 | 5 | 1.75 | 10 | 18.2 | RNA |
| **ATP1A1** | P05023 | Sodium/potassium-transporting ATPase subunit alpha-1 | 5 | 1.72 | 5 | 11.7 | Cytoskeleton |
| **LAMC1** | P11047 | Laminin subunit gamma-1 | 4 | 1.67 | 3 | 102.1 | Other/Unknown |
| **CTSC** | P53634 | Dipeptidyl peptidase 1 | 4 | 1.66 | 8 | 32.2 | Other/Unknown |
| **HAX1** | O00165 | HCLS1-associated protein X-1 | 6 | 1.60 | 12 | 35.7 | Cytoskeleton |
| **HSPA8** | P11142 | Heat shock cognate 71 kDa protein | 7 | 1.59 | 9 | 46.3 | Protein Folding |
| **CTNNB1** | P35222 | Catenin beta-1 | 4 | 1.59 | 4 | 22.6 | Signal Transduction |
| **MATR3** | P43243 | Matrin-3 | 4 | 1.57 | 4 | 40.2 | RNA |
| **PSMD2** | Q13200 | 26S proteasome non-ATPase regulatory subunit 2 | 5 | 1.53 | 8 | 67.4 | Other/Unknown |
| **CDKN2AIPNL** | Q96HQ2 | CDKN2AIP N-terminal-like protein | 2 | 1.53 | 3 | 4.2 | Other/Unknown |
| **HNRNPL** | P14866 | Heterogeneous nuclear ribonucleoprotein L | 6 | 1.52 | 7 | 45.3 | RNA |
| **DPP4** | P27487 | Dipeptidyl peptidase 4 | 1 | 1.51 | 3 | 14.3 | Other/Unknown |

^a^UniProt accession number, ^b^DENV/Mock enrichment ratio calculated by MaxQuant, ^c^Number of peptides used to calculate the DENV/Mock TUX-MS ratio, ^d^The variance in the DENV/Mock TUX-MS ratio, calculated as the coefficient of variation in peptide ratio counts.

**References**

1. Yocupicio-Monroy, R.M., Medina, F., Reyes-del Valle, J. and del Angel, R.M. (2003) Cellular proteins from human monocytes bind to dengue 4 virus minus-strand 3' untranslated region RNA. *Journal of virology*, **77**, 3067-3076.

2. Khadka, S., Vangeloff, A.D., Zhang, C., Siddavatam, P., Heaton, N.S., Wang, L., Sengupta, R., Sahasrabudhe, S., Randall, G., Gribskov, M. *et al.* (2011) A physical interaction network of dengue virus and human proteins. *Molecular & cellular proteomics : MCP*, **10**, M111 012187.

3. Limjindaporn, T., Wongwiwat, W., Noisakran, S., Srisawat, C., Netsawang, J., Puttikhunt, C., Kasinrerk, W., Avirutnan, P., Thiemmeca, S., Sriburi, R. *et al.* (2009) Interaction of dengue virus envelope protein with endoplasmic reticulum-resident chaperones facilitates dengue virus production. *Biochemical and biophysical research communications*, **379**, 196-200.

4. Gomila, R.C., Martin, G.W. and Gehrke, L. (2011) NF90 binds the dengue virus RNA 3' terminus and is a positive regulator of dengue virus replication. *PloS one*, **6**, e16687.

5. De Nova-Ocampo, M., Villegas-Sepulveda, N. and del Angel, R.M. (2002) Translation elongation factor-1alpha, La, and PTB interact with the 3' untranslated region of dengue 4 virus RNA. *Virology*, **295**, 337-347.

6. Anwar, A., Leong, K.M., Ng, M.L., Chu, J.J. and Garcia-Blanco, M.A. (2009) The polypyrimidine tract-binding protein is required for efficient dengue virus propagation and associates with the viral replication machinery. *The Journal of biological chemistry*, **284**, 17021-17029.

7. Agis-Juarez, R.A., Galvan, I., Medina, F., Daikoku, T., Padmanabhan, R., Ludert, J.E. and del Angel, R.M. (2009) Polypyrimidine tract-binding protein is relocated to the cytoplasm and is required during dengue virus infection in Vero cells. *The Journal of general virology*, **90**, 2893-2901.

8. Mishra, K.P., Shweta, Diwaker, D. and Ganju, L. (2012) Dengue virus infection induces upregulation of hn RNP-H and PDIA3 for its multiplication in the host cell. *Virus research*, **163**, 573-579.

9. Paranjape, S.M. and Harris, E. (2007) Y box-binding protein-1 binds to the dengue virus 3'-untranslated region and mediates antiviral effects. *The Journal of biological chemistry*, **282**, 30497-30508.

10. Brunetti, J.E., Scolaro, L.A. and Castilla, V. (2015) The heterogeneous nuclear ribonucleoprotein K (hnRNP K) is a host factor required for dengue virus and Junin virus multiplication. *Virus research*.

11. Chang, C.J., Luh, H.W., Wang, S.H., Lin, H.J., Lee, S.C. and Hu, S.T. (2001) The heterogeneous nuclear ribonucleoprotein K (hnRNP K) interacts with dengue virus core protein. *DNA and cell biology*, **20**, 569-577.

12. Chen, X., Xia, J., Zhao, Q., Wang, Y., Liu, J., Feng, L., He, J. and Zhang, P. (2015) Eukaryotic initiation factor 4AI interacts with NS4A of Dengue virus and plays an antiviral role. *Biochemical and biophysical research communications*.

13. Ward, A.M., Bidet, K., Yinglin, A., Ler, S.G., Hogue, K., Blackstock, W., Gunaratne, J. and Garcia-Blanco, M.A. (2011) Quantitative mass spectrometry of DENV-2 RNA-interacting proteins reveals that the DEAD-box RNA helicase DDX6 binds the DB1 and DB2 3' UTR structures. *RNA biology*, **8**, 1173-1186.

14. Phillips, S.L., Soderblom, E.J., Bradrick, S.S. and Garcia-Blanco, M.A. (2016) Identification of Proteins Bound to Dengue Viral RNA In Vivo Reveals New Host Proteins Important for Virus Replication. *MBio*, **7**.

15. Kurosu, T., Chaichana, P., Yamate, M., Anantapreecha, S. and Ikuta, K. (2007) Secreted complement regulatory protein clusterin interacts with dengue virus nonstructural protein 1. *Biochemical and biophysical research communications*, **362**, 1051-1056.

16. Lei, Y., Huang, Y., Zhang, H., Yu, L., Zhang, M. and Dayton, A. (2011) Functional interaction between cellular p100 and the dengue virus 3' UTR. *The Journal of general virology*, **92**, 796-806.
