# Supplementary material for: Identification of RNA Binding Proteins Associated with Dengue Virus RNA in Infected Cells Reveals Temporally Distinct Host Factor Requirements
Source: PLoS Negl Trop Dis. 2016 Aug 24;10(8):e0004921. doi: 10.1371/journal.pntd.0004921 (PMC4996428; doi:10.1371/journal.pntd.0004921)
Supplement: S1 Methods — (DOC) [file pntd.0004921.s003.doc]

**Supplementary Methods**

**Quantitative mass spectrometry-based proteomic analysis of RNA-interacting proteins.** Protein eluates and their respective mixed light/heavy input lysates were subjected to in-solution enzymatic digestion using a filter-aided sample preparation approach [1], as we described in [2]. Briefly, eluates were adjusted with 4X NuPAGE sample buffer (lacking loading dye and glycerol) to a final 1X concentration of 247 mM Tris, pH 8.5, 2% LDS, and 0.51 mM EDTA. Proteins were reduced with 100 mM DTT at 70°C for 5 min, then transferred to Amicon-500 ultrafiltration devices. Samples were washed twice with UB buffer (8 M urea in 0.1 M TrisHCl, pH 8.0) followed by centrifugation at 14,000 x g for 10 min at 20°C. Proteins were then alkylated in UB buffer containing 50 mM iodoacetamide at RT for 30 min. Samples were washed thrice with UB buffer, then once with 50 mM ammonium bicarbonate (ABC). 100 µL of 50 mM ABC containing 5 µg/ml trypsin was added to the samples and incubated at 37°C overnight. The samples were washed twice with 25 µL of water, and the filtrate containing the tryptic peptides was recovered. The peptides from the mixed input lysates were desalted over C_18_ StageTips, while the peptides from the eluate sample were desalted and separated into four fractions over stacked C_18_-strong cation exchange (SCX) StageTips, as previously described [3] with some modifications. Specifically, pH-based SCX fractionation of peptides was achieved using sequential application of 30 µl of the following volatile ammonium salt buffers: 50 mM ammonium formate/20% acetonitrile (ACN), 50 mM ammonium acetate/20% ACN, 50 mM ammonium bicarbonate/20% ACN, and 0.1% ammonium hydroxide/20% ACN. Fractions were concentrated to near dryness, resuspended in 10 µl of 0.1% formic acid, and analyzed by nLC-MS/MS.

Briefly, peptides (4 µl) were separated by reverse phase chromatography-nLC (Acclaim PepMap RSLC, 1.8 μm x 75 μm x 50 cm) at a flow rate of 250 nL/min over a 90 min ACN gradient from 4 – 40% mobile phase B (A, 0.1% FA; B, 97% ACN in 0.1% FA) and ionized by ESI directly into the mass spectrometer (LTQ Orbitrap Velos). The mass spectrometer was operated in a data-dependent acquisition mode. Each acquisition cycle comprised a single full-scan mass spectrum (m/z = 350-1700) in the orbitrap (r = 60,000 at m/z = 400) followed by CID MS/MS of the top 20 most abundant ions, with dynamic exclusion enabled.

**Quantitative mass spectrometry-based proteomic analysis of DENV-infected cells**. Light (DENV-infected) and heavy-labeled (mock) cell pellets were lysed in 100 mM ABC containing 5% sodium deoxycholate at 95°C to ensure denaturation and virus inactivation. The lysate protein concentrations were determined by the BCA assay and mixed in equal protein amounts (100 µg total). Proteins were reduced and alkylated simultaneously with TCEP (25 mM)/chloroacetamide (25 mM) at 70°C for 20 min, then quenched with cysteine (25 mM). The mixed lysates were diluted 1:4 with high purity water and trypsin was added in 1:50 enzyme:protein ratio, followed by overnight incubation at 37°C. After digestion, peptides were purified using an acidic phase transfer method. An equal volume of ethyl acetate was added to the samples, then adjusted to 0.5% trifluoroacetic acid, as previously [4]. Peptides in the aqueous fraction were recovered, then desalted, and separated into three fractions over SDB-RPS StageTips [5]. Each SDB-RPS fraction was further separated over C_18_-SCX StageTips, as described above, resulting in 12 total fractions. Each fraction was analyzed in duplicate by nLC-MS/MS as described above, except peptide fractions were separated over a 3 hour ACN gradient from 4 – 35% mobile phase buffer B.

**Proteomic data analysis**.  The qTUX-MS, its respective mixed input lysate, and the whole cell SILAC instrument raw data were separately processed using the MaxQuant software (ver. 1.5.3.8), configured with default settings, except for the following experiment-specific parameters. A multiplicity of two (light and heavy) was specified, defining the heavy-labelled amino acids as Lys6 and Arg10. MS/MS spectra were searched against the UniProt human sequence database (release 2013-08) appended with UniProt DENV-2 polyprotein sequences and common experimental contaminants (20, 382 sequences). Variable specified modifications were N-terminal acetylation and methionine oxidation, with a static modification of cysteine carbamidomethylation. The peptide-spectrum match (PSM) and protein false discovery rates were controlled to < 1%, based on a reversed sequence database search. Relative protein quantification, based on peptide SILAC ratios, was performed with “Match between Runs” enabled. The re-quantify option was only enabled for the qTUX-MS data. The proteinGroups.txt files were imported to Perseus (ver. 1.5.2.6) for additional data processing. Proteins reflecting environmental contaminants and nuclear histones were removed. Protein identifications were retained if the number of quantified peptides (ratio counts) were at least three in the qTUX-MS data and two in the whole cell datasets. qTUX-MS light/heavy ratios, which reflect the relative enrichment with DENV RNA, were normalized by the median light/heavy ratio of proteins identified in the qTUX-MS input lysates. Proteins with ≥ 1.5-fold ratio enrichment were considered candidate DENV RNA binding proteins. The mass spectrometry proteomics data have been deposited to the ProteomeXchange Consortium via the PRIDE [6] partner repository with the dataset identifier PXD003593.

1. Wisniewski JR, Zougman A, Nagaraj N, Mann M. Universal sample preparation method for proteome analysis. Nature methods. 2009;6(5):359-62. doi: 10.1038/nmeth.1322. PubMed PMID: 19377485.

2. Joshi P, Greco TM, Guise AJ, Luo Y, Yu F, Nesvizhskii AI, et al. The functional interactome landscape of the human histone deacetylase family. Molecular systems biology. 2013;9:672. Epub 2013/06/12. doi: 10.1038/msb.2013.26. PubMed PMID: 23752268.

3. Ishihama Y, Rappsilber J, Mann M. Modular stop and go extraction tips with stacked disks for parallel and multidimensional Peptide fractionation in proteomics. J Proteome Res. 2006;5(4):988-94. doi: 10.1021/pr050385q. PubMed PMID: 16602707.

4. Masuda T, Tomita M, Ishihama Y. Phase transfer surfactant-aided trypsin digestion for membrane proteome analysis. J Proteome Res. 2008;7(2):731-40. doi: 10.1021/pr700658q. PubMed PMID: 18183947.

5. Kulak NA, Pichler G, Paron I, Nagaraj N, Mann M. Minimal, encapsulated proteomic-sample processing applied to copy-number estimation in eukaryotic cells. Nature methods. 2014;11(3):319-24. doi: 10.1038/nmeth.2834. PubMed PMID: 24487582.

6. Vizcaino JA, Csordas A, del-Toro N, Dianes JA, Griss J, Lavidas I, et al. 2016 update of the PRIDE database and its related tools. Nucleic Acids Res. 2016;44(D1):D447-56. doi: 10.1093/nar/gkv1145. PubMed PMID: 26527722; PubMed Central PMCID: PMCPMC4702828.
