# Supplementary figures and images for: Identification of RNA Binding Proteins Associated with Dengue Virus RNA in Infected Cells Reveals Temporally Distinct Host Factor Requirements
Source: PLoS Negl Trop Dis. 2016 Aug 24;10(8):e0004921. doi: 10.1371/journal.pntd.0004921 (PMC4996428; doi:10.1371/journal.pntd.0004921)

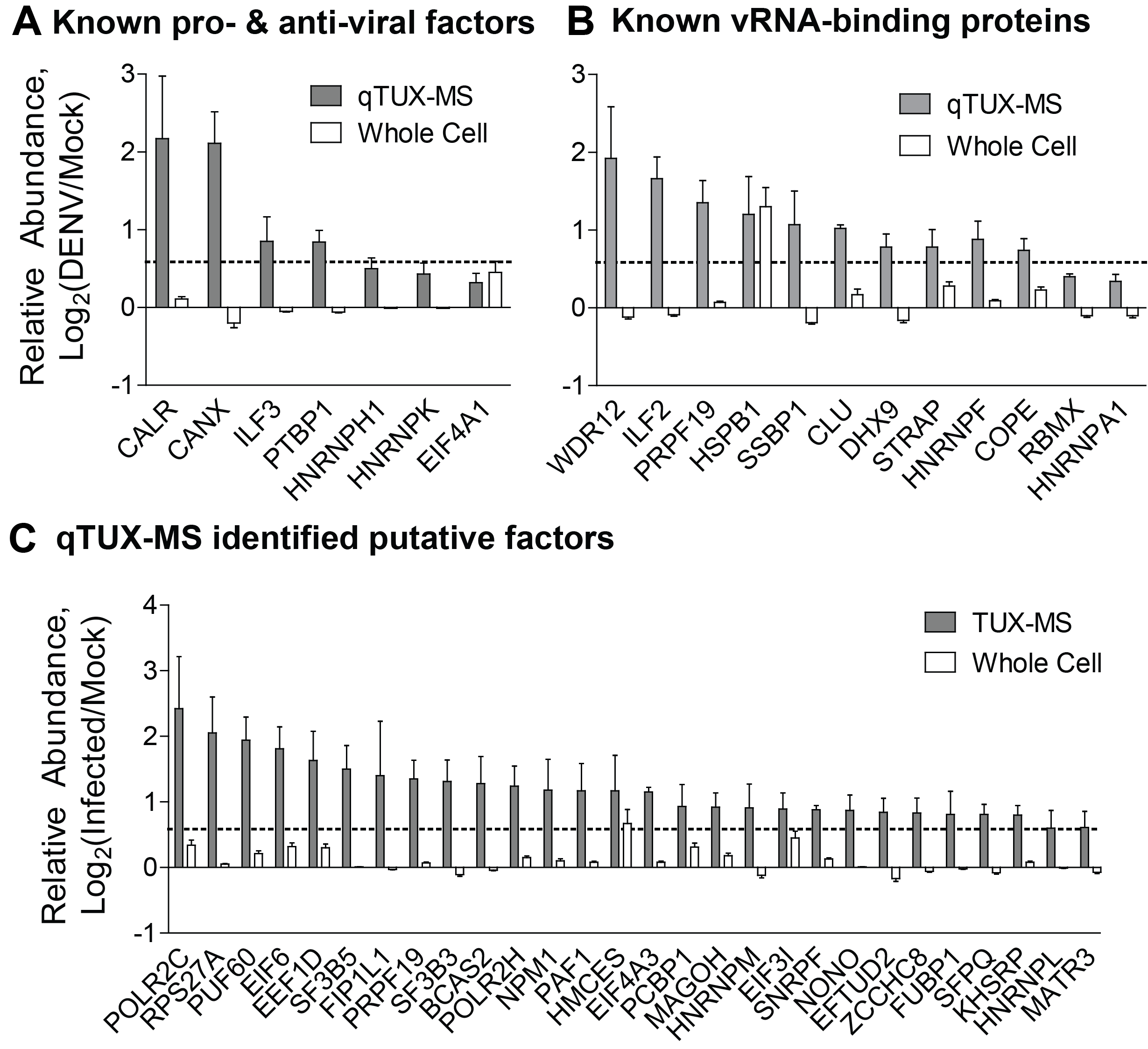

Supplement: S1 Fig — The relative log2 abundance of each protein (indicated by its gene symbol) was compared for the qTUX-MS (grey bars) and whole cell analysis (white bars) for (A) known DENV pro- and anti-viral factors, (B) known DENV vRNA-binding proteins, and (C) qTUX-MS identified putative factors. The error bars represent the protein ratio variability, calculated from individual light/heavy peptide ratios. Dashed line indicates the 1.5-fold enrichment threshold. (TIF) [file pntd.0004921.s005.tif]

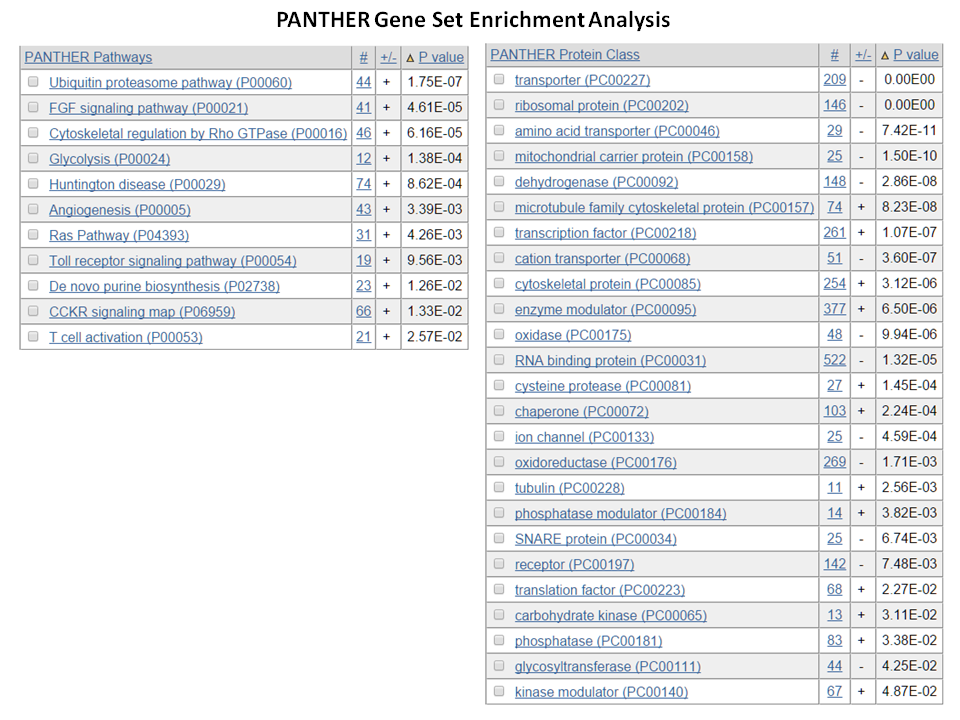

Supplement: S2 Fig — The genes and relative expression ratios of host proteins quantified from Huh7.5 whole cell lysates (N = 4907) were submitted to PANTHER gene enrichment using the default settings (www.pantherdb.org). The enrichment of gene functions was performed using the PANTHER “Pathways” and “Protein Class” ontologies. Only the statistically significant ontology terms are displayed, along with the number of genes annotated in that ontology, whether that set of genes was systematically up (+) or down (-) regulated, and the corresponding p-value. (TIF) [file pntd.0004921.s006.tif]

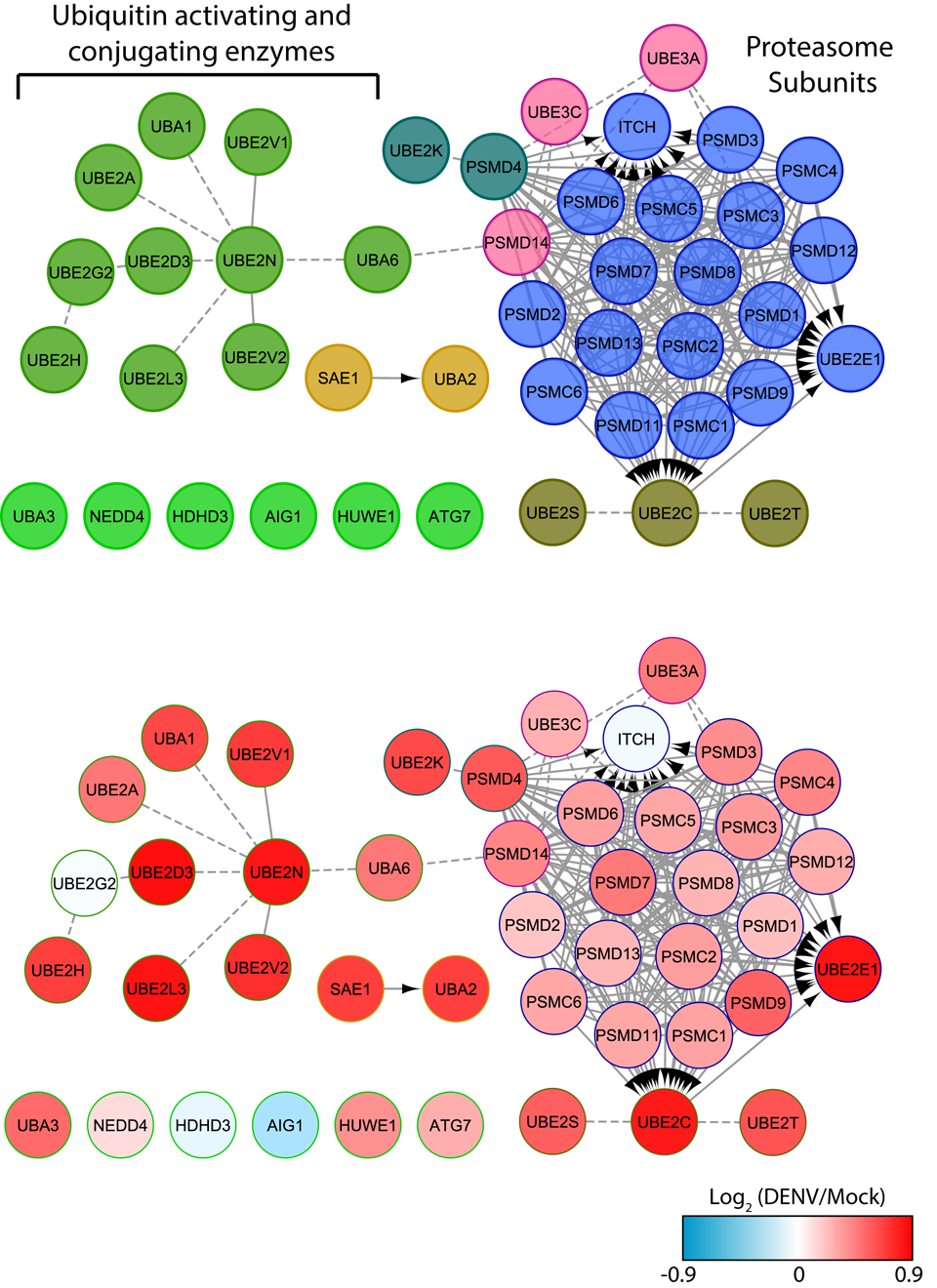

Supplement: S3 Fig — PANTHER gene enrichment annotated 44 proteins to the Ubiquitin-Proteasome Pathway, which on average were significantly up-regulated upon DENV infection. The corresponding genes were analyzed by the Reactome Functional Interaction (FI) Cytoscape plug-in. Sub-networks (≥ 2 genes per network) were assembled with nodes colors representing functional clustering by Reactome FI (top) or log2 DENV/Mock relative abundance changes (bottom). Network edges represent Reactome functional interactions:–, protein complex; →, activating;–|, inhibiting; —, predicted. Clusters were labeled with protein functions/activities representative of the majority of proteins within each cluster. (TIF) [file pntd.0004921.s007.tif]

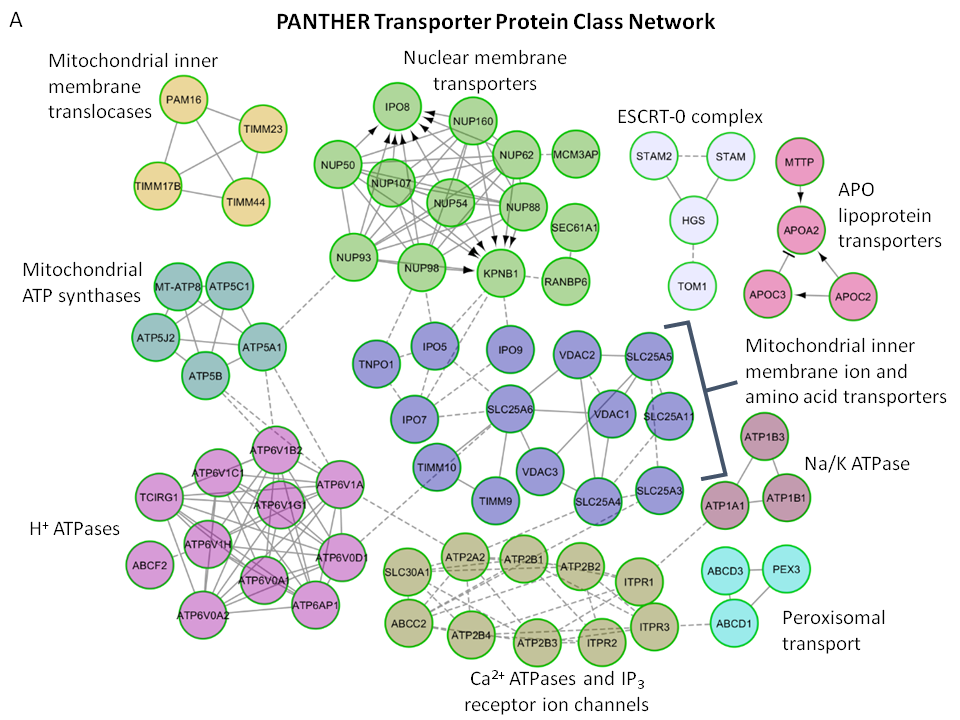

Supplement: S4 Fig — PANTHER gene enrichment classified 209 proteins annotated as transporters, which on average were significantly down regulated upon DENV infection. The corresponding genes were analyzed by the Reactome Functional Interaction (FI) Cytoscape plug-in. Four distinct sub-networks (≥ 3 genes per network) were assembled containing 71 out of the 209 genes. Nodes colors represent functional clustering by Reactome FI. Network edges represent Reactome functional interactions:–, protein complex; →, activating;–|, inhibiting; —, predicted. Clusters were labelled with the transporter classes and/or activities represented by the majority of proteins within that cluster. (TIF) [file pntd.0004921.s008.tif]

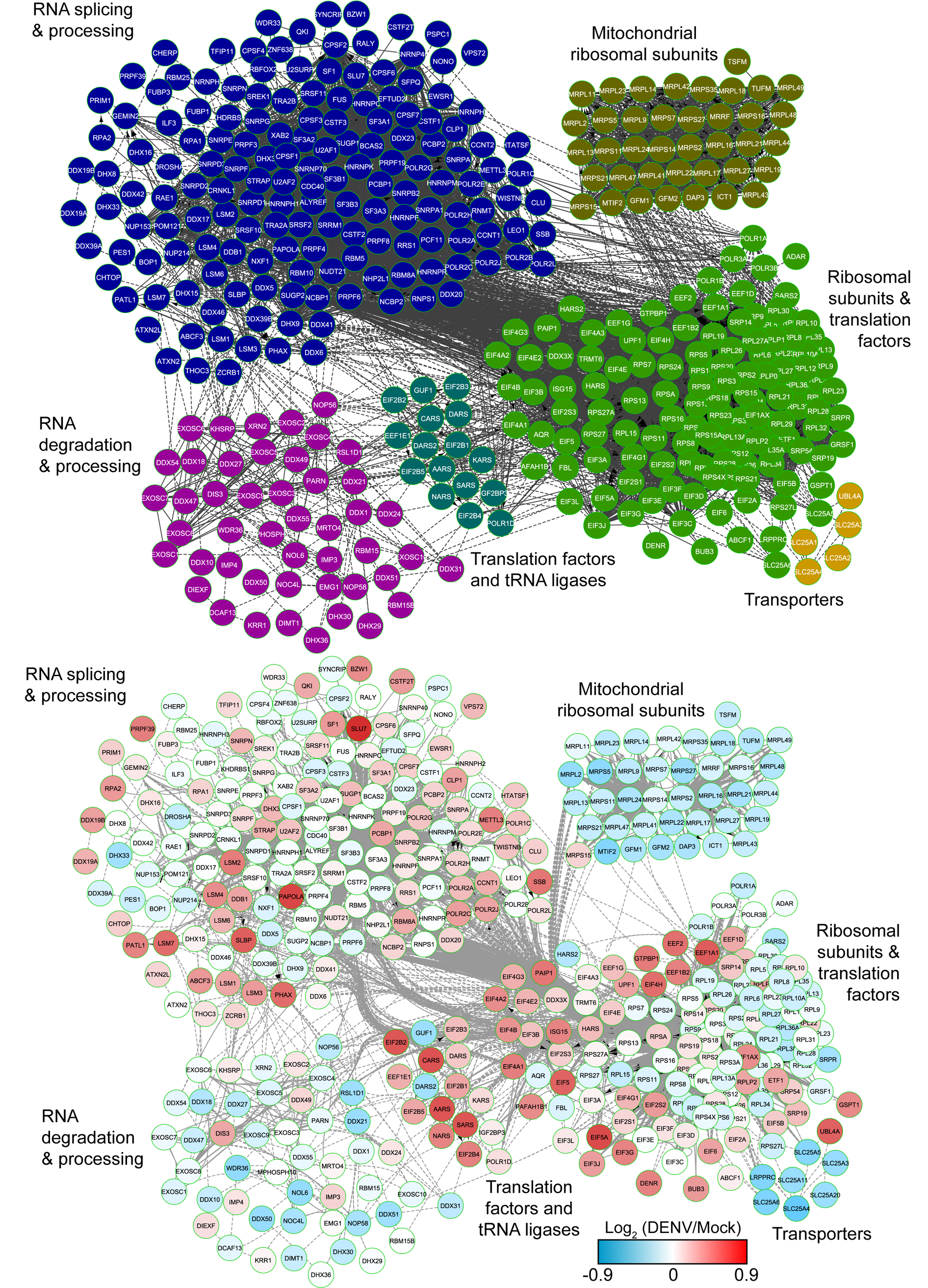

Supplement: S5 Fig — PANTHER gene enrichment classified 523 proteins annotated as RNA binding proteins, which on average were significantly down regulated upon DENV infection. The corresponding genes were analyzed by the Reactome Functional Interaction (FI) Cytoscape plug-in. Six sub-networks (≥ 3 genes per network) were assembled with modes colors representing functional clustering by Reactome FI (top) or log2 DENV/Mock relative abundance changes (bottom). Network edges represent Reactome functional interactions:–, protein complex; →, activating;–|, inhibiting; —, predicted. Clusters were labelled with the transporter classes and/or activities represented by the majority of proteins within that cluster. (TIF) [file pntd.0004921.s009.tif]

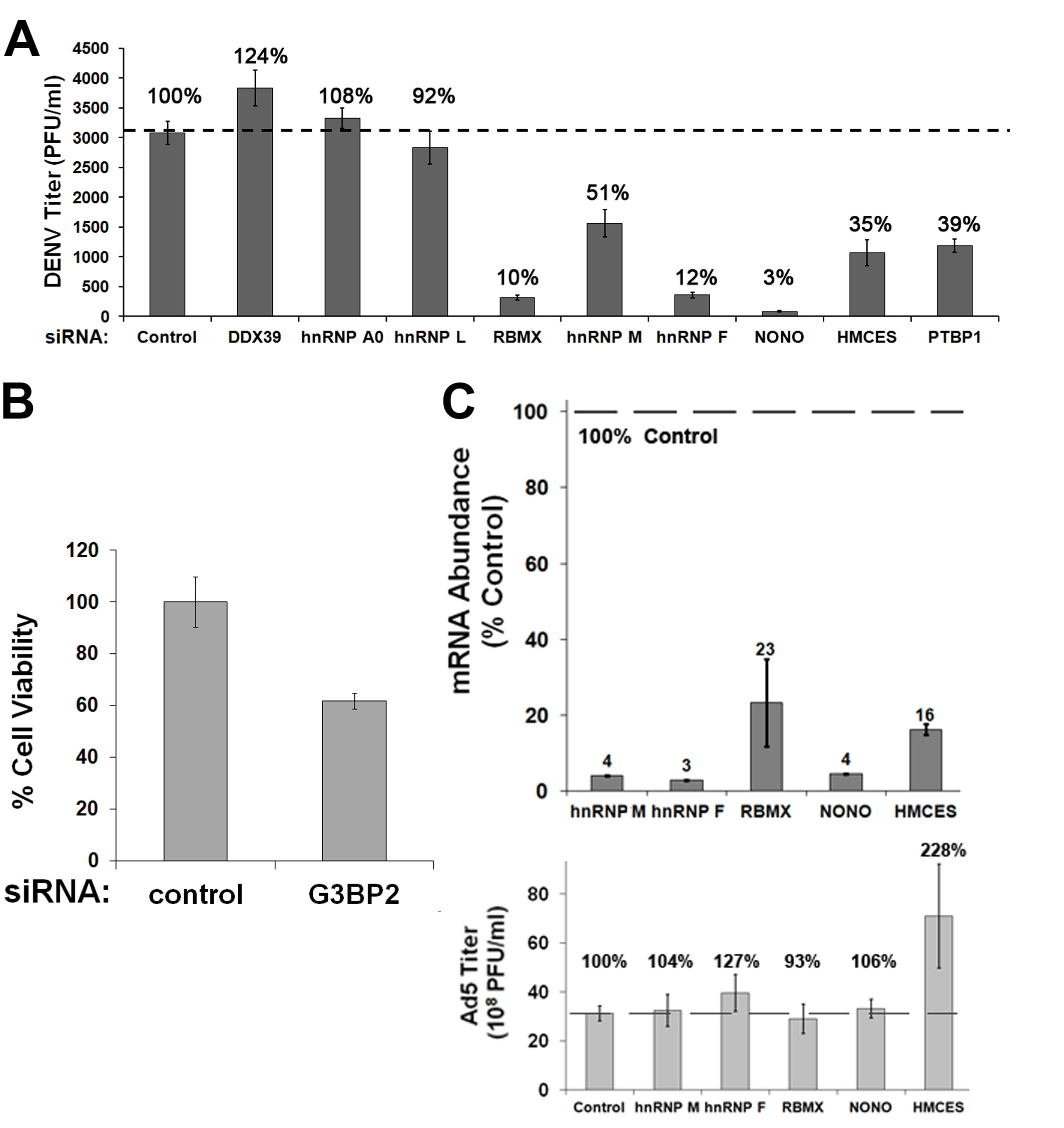

Supplement: S6 Fig — (A) HeLaUPRT cells were transfected with either control or specific siRNAs. 24 hours post transfection cells were counted and seeded (4 X 105 cells/well) in 6-well plates in triplicates. 48 hours post transfection cells were infected with DENV2 at MOI 0.1 and virus released to the media collected 40 hours post infection. DENV2 titers were measured using plaque assays. The bars represent average values from triplicate, a standard error is reported. The representative data from one of at least two independent experiments is shown. (B) To verify sensitivity of the MTT assay, we performed siRNA knockdown of G3BP2, which is known to bind DENV and was previously shown to affect cell viability [37, 75]. Relative viability of non-infected cells was measured using an MTT assay (Invitrogen) and represented exactly as described in Fig 5C. Cell viability or proliferation is decreased by knockdown of G3BP2 compared with control siRNA (p<0.01). siRNA transfection was performed as described in the methods section using previously published siRNA sequence [37]. In cells treated with G3BP2-specific siRNA but not control siRNA, G3BP2 protein was knocked down to the levels undetectable by western analysis using antibodies against G3BP2 (Abcam, ab86135). (C). HeLaUPRT cells were seeded 4.0 X 105 cells in 60 mm plates and transfected with either control or specific siRNAs. 24 hours post transfection cells were infected with Ad5 at MOI 0.1 and collected 30 hours post infection. siRNA knockdown efficiency was determined by measuring respective mRNA levels in comparison to β-actin mRNA abundance as described in the Experimental Section and reported relative to control siRNA transfection (upper panel). Ad5 titers were determined using plaque assays on 911 cells. The bars represent average values from triplicate, a standard error is reported. The representative data from one of at least three independent experiments is shown. (TIF) [file pntd.0004921.s010.tif]
